# Citation gaming induced by bibliometric evaluation: A country-level comparative analysis

**DOI:** 10.1371/journal.pone.0221212

**Published:** 2019-09-11

**Authors:** Alberto Baccini, Giuseppe De Nicolao, Eugenio Petrovich

**Affiliations:** 1 Department of Economics and Statistics, University of Siena, Siena, Italy; 2 Department of Electrical, Computer and Biomedical Engineering, University of Pavia, Pavia, Italy; Max Planck Society, GERMANY

## Abstract

It is several years since national research evaluation systems around the globe started making use of quantitative indicators to measure the performance of researchers. Nevertheless, the effects on these systems on the behavior of the evaluated researchers are still largely unknown. For investigating this topic, we propose a new inwardness indicator able to gauge the degree of scientific self-referentiality of a country. Inwardness is defined as the proportion of citations coming from the country over the total number of citations gathered by the country. A comparative analysis of the trends for the G10 countries in the years 2000-2016 reveals a net increase of the Italian inwardness. Italy became, both globally and for a large majority of the research fields, the country with the highest inwardness and the lowest rate of international collaborations. The change in the Italian trend occurs in the years following the introduction in 2011 of national regulations in which key passages of professional careers are governed by bibliometric indicators. A most likely explanation of the peculiar Italian trend is a generalized strategic use of citations in the Italian scientific community, both in the form of strategic author self-citations and of citation clubs. We argue that the Italian case offers crucial insights on the constitutive effects of evaluation systems. As such, it could become a paradigmatic case in the debate about the use of indicators in science-policy contexts.

## Introduction

Starting from the late 1980s, several European and extra-European countries implemented national systems to monitor, assess, and evaluate the research performance of their scientific workforce [1, 2]. One of the key features of such research evaluation systems is the focus on quantitative indicators (metrics) as crucial science policy tools [3]. Accordingly, in the last years, several scientometric indicators, based on publications or citations (or on a combination of both, such as the *h-index*), have increasingly appeared in the academic evaluation systems, alongside with the traditional peer-review-based procedures.

The use of these indicators in the evaluation of research performance has generated a heated debate in the scientific community. The advocates argue that scientometric measures are not only more objective than the peer-review [4]; they would also improve both the quantity and the quality of the scientific production [5, 6]. This would occur because the indicators are integrated within a system of incentives that rewards the achievement of the scientometric targets set by the evaluation system [7]. On the other hand, critics claim that the same mechanisms that are designed to improve the research performance create at the same time room for strategic behaviors [8]. For instance, when productivity is positively rewarded, the number of publications become a goal that can be pursued not only by positive behaviors (doing more research), but also by opportunistic strategies (e.g., slicing one scientific work into multiple publications) [9, 10]. Analogously, when citations become a goal, the “citation game” starts [11]. Criticisms themselves have been challenged: for instance, Butler’s conclusions about the Australian case have been widely discussed [12]. A mediating position is represented by scholars proposing a “responsible use” of metrics. According to this approach, research metrics can provide valuable insights on the research performance, granted that they are carefully designed in order to avoid unintended consequences. Thus, a distillation of best practices has been proposed for improving the use of metrics in research assessment [13].

Recently, the idea that the consequences of the use of indicators on the behavior of researchers can be easily sorted between the intended and the unintended ones, has been questioned as too simplistic [14, 15]. Instead, the notion of “constitutive effects” has been advanced to capture the way in which the indicators act on the researchers [16]. Within this new framework, indicators are conceived as shaping the activity of research deeply and at different levels, from the citation habits to the research agenda, redefining at the same time key evaluative terms such as research quality [17]. They become crucial actors in the “epistemic living spaces” of academic researchers [18] and researchers begin to “think with indicators” pervasively [19].

The main constitutive effects of the indicators described in the literature can be grouped into three main types: i) Goal-displacement: scoring high on the indicators becomes a target in itself, that is to be achieved also by *gaming* the system [20, 21]; ii) Risk avoidance: highly innovative, not mainstream, and interdisciplinary research topics are avoided because they could do not score well on indicators that tend to reward more traditional research programmes [19, 22–26]; iii) Task reduction: when academic activities such as teaching and public engagement are not rewarded, academics tend to avoid them to concentrate only on publishable academic research [27–29].

Although these effects have been highly debated, until recently the evidence of their occurrence has been mainly anecdotal. It is only in the last years that the methodical empirical study of such effects has been undertaken [14, 22]. In the present paper, we aim to advance the knowledge on this topic by focusing on the case of Italy. Among European and extra-European countries, Italy is the only one in which some key career passages of scientific researchers are entirely regulated by rules based on bibliometric indicators (except for the scholars in the Social Sciences and Humanities, see next section). Thus, Italy is ideally suited to studying the response of researchers to the use of metrics in research evaluation.

In particular, we will investigate whether Italian scientists have pervasively adopted a strategic use of citations in order to boost their indicators. By “pervasively”, we mean that the effect of this behavior should be visible *in the great majority of scientific fields, at the national level*. As we will highlight in the Conclusion, the Italian case provides important insights on the constitutive effects of evaluation systems in general.

The rest of the paper is organized as follows. In the next two sections, the specificity of the Italian case is explained and the literature dealing with self-citing strategic behaviors is reviewed. Next, a new “inwardness” indicator is introduced that is sensitive to collective strategic citation behaviors at a country level. In the Data section, the procedure for retrieving the data is described, while the main findings are presented in the Results section. In the Discussion, after examining alternative explanations, it is argued in favor of the emergence of a collective strategic behavior devised to meet the demands of the evaluation system. In the Conclusions, some general lessons from the Italian case are drawn.

## The Italian case

In 2010, the Italian university system underwent a wide process of reformation, regulated by the Law 240/2010. The reform created the Agency for the Evaluation of the University and Research (ANVUR), a centralized agency whose main task is the monitoring and the evaluation of the Italian research system. The Agency started in 2011 a research assessment exercise called VQR, relative to the period 2004-2010. A second research assessment exercise was started in 2015, relative to the period 2011-2014. In both exercises, the evaluation of submitted articles was largely based on the automatic or semi-automatic use of algorithms fed by citation indicators [30] while other research outputs, such as books, were evaluated by peer reviews.

The reform modified also the recruitment and advancement system for university professors by introducing the National Scientific Habilitation (ASN). Both for hiring and promotion, having obtained the ASN has become mandatory for applying to academic positions. The bibliometric rules rely on three indicators. For the hard sciences, life sciences, and engineering, the indicators considered by ANVUR are the number of journal articles, the number of citations, and the h-index. For the social science and humanities, the indicators are the number of research outputs, the number of monographs, and the number of papers published in “class A” journals. At each new round of habilitation, ANVUR calculates for each of these indicators the “bibliometric thresholds” that the candidates must overcome to achieve the ASN. For the first edition of the ASN the national rules were defined in the Ministerial Decree 7 June 2012 n. 76. http://attiministeriali.miur.it/media/192901/dm_07_06_12_regolamento_abilitazione.pdf. ANVUR defined the thresholds used for the first edition of the ASN: https://web.archive.org/web/20190207112821/http://www.anvur.it/attivita/asn/asn-2012-2013/indicatori-e-relative-mediane/. Candidates whose indicators do not overcome two thresholds out of three cannot be habilitated (exceptions were possible in specific circumstances only in the first edition, ASN 2012). When first introduced, the thresholds were stated to be the median values of the indicators of the permanent academic staff holding that position (associate or full professor). To make and example, in order to obtain a full professor habilitation, the candidate was required to score better than half of the current full professors in two indicators out of three. Applicants overcoming the fixed thresholds are then evaluated by a committee composed by five referees who are in charge of the final decision about attributing habilitation.

Note that the focus on indicators is not confined to the national procedures but “trickles down” to the university committees in charge of recruiting and promotion that are required to take into account production and citation metrics when they evaluate and rank the habilitated applicants. Finally, also the members of both the national habilitation and the local recruitment committees are required to overcome bibliometric thresholds.

In sum, in Italy, starting from 2011, bibliometric indicators have gained a central role not only in the national research assessment but in the entire body of the recruitment procedures. A remarkable peculiarity of the Italian system is that the indicators based on citations, used both in the habilitation procedure and in the research evaluation exercise, are calculated *by including self-citations*. Thus, researchers can increase their indicators just by self-citing their own work.

Anecdotal evidence of the adoption of strategic behaviors in the form of author self-citations has been presented by Baccini [31]. Two recent studies have documented more thoroughly the rise of opportunistic behaviors in response to the ASN rules. Seeber et al. has analyzed how the use of self-citations in four Italian research areas changed after the introduction of the habilitation procedure. They have found that scientists in need of meeting the thresholds (i.e., those looking for habilitation as a prerequisite for tenure-track or promotion to full professor) did increase significantly their self-citations after 2010 [32]. Scarpa et al. focused on the Italian engineering area and found an anomalous peak in the self-citations rate (i.e., the number of self-citations to the total number of citations) in correspondence of the second round of the habilitation procedure, in 2013 [33]. Even if the aforementioned studies have highlighted some recent behavior changes by Italian scientists, they did not address a subtler form of strategic behavior, the one based on the so-called citation clubs or citation cartels.

## Strategic behaviors, country self-citations, and the inwardness indicator

A citation club is an informal structure in which citations are strategically exchanged among its members to boost the respective citation scores [34–36]. Citation clubs are difficult to spot, especially when their members exchange citations but are not co-authors. Indeed, if we only examine the self-citation rates of the individual members, we would not spot any anomaly, in so far as they keep their individual self-citations under control (i.e., they do not cite disproportionately their own work). Thus, a well-concealed citation club is invisible if monitoring is limited to individual self-citations [37, 38]. If we consider a group of scholars, the citation club becomes visible as it increases the citation traffic internal to the group (group self-citations). Obviously, groups of scholars may be individuated in many ways and in different social networks. A most natural example may be a group of scholars that are not directly co-authors but at a relatively small distance in a co-authorship network. However, a citation club may also thrive on an interlocking editorship network [39, 40], in which case citations are exchanged between scholars serving as editors in the same set of journals. Or, again, the citation club may be rooted at an institutional level (universities or departments). In all these cases, although it is possible to record the citation traffic inside the citation club, it is nonetheless impossible to distinguish the citations generated as a normal by-product of the research activity from those resulting from strategic behaviours.

Along this rationale, the key idea of this paper is that a sudden and strong increase of strategic citations internal to a country is going to affect in a visible way self-citations recorded at country level. Such occurrence may be spotted by a macro level analysis, without the need of documenting the existence of clubs, whatever defined, and of a criterion to distinguish between types of citations. Hence, hereafter the focus is on *country self-citations*, a not much studied form of self-citation [41]

A country self-citation occurs whenever the set of the countries of the authors of the citing publication and the set of the countries of the authors of the cited publication are not disjoint, that is, if these two sets share at least one country [42, 43]. Notably, any citation exchanged within a citation club formed by researchers working in the same country is counted as country self-citations, even when it is not an author self-citation.

Thus, considering that most of the standard author self-citations are country self-citations too (the only exception being authors that changed their country between the citing and the cited publication), by analyzing the country self-citations, we can capture both the “classic” strategy based on author self-citations, and the “elaborated” one based on citation clubs.

It is very important to underline that country self-citations are not always generated by citation clubs, just as not all author self-citations originate from gaming purposes. The literature on author self-citations agrees on the fact that a certain amount of them is a normal byproduct of the scientific communication. There are many perfectly legitimate reasons for citing one’s own works, such as building on previously obtained results, avoiding repetition, and so on [44–46]. By the same token, it is normal that a country has an internal exchange of citations amongst its researchers insofar the knowledge produced by the country is used (i.e., cited) by the same country’s scientific staff. Moreover, in the research fields that are characterized by a national focus (e.g., some areas in the Social Science and Humanities), it is normal to expect a larger number of country self-citations.

Consider also that international collaboration positively affects the number of country self-citations. In fact, the more a country collaborates with other countries, the higher will be the number of country self-citations. Take for instance a paper authored in collaboration by Italy and France. Any future citation to that paper coming from an Italian-authored or a French-authored publication will count as a country self-citation for both Italy and France, since the citing and the cited publication will share at least one country of affiliation.

In sum, the country self-citations are not *per se* a sign of strategic behavior. The level of self-citations of a country depends both on the internal exchange of knowledge within a country and the amount of international collaboration. Nonetheless, if the researchers of a single country initiate strategic behaviors in order to boost their citations, this is likely to produce an *anomalous increase* of country self-citations compared to the other countries. Thus, to detect the strategic behaviors, one has to focus on the *changes* in the country self-citations over time, rather than on their absolute value.

In order to obtain a normalized measure of country self-citations, we introduce a simple indicator of “inwardness”. For a given year and a country *c*, the inwardness is defined as the percentage ratio between the total number of country self-citations (*S*_*c*_) and the total number of citations (*C*_*c*_) of that country:

$$I_{c}=\frac{S_{c}}{C_{c}}\times 100$$ (1)

The minimum value of the inwardness indicator is *I*_*c*_ = 0 when a country has no self-citations; and the maximum is *I*_*c*_ = 100 when a country has self-citations only, that is *S*_*c*_ = *C*_*c*_.

It is easy to show that the inwardness indicator is a variant of the Relative Citation Impact (*RCI*) of a country. The *RCI* is defined by May [47] as the ratio between the average citation per paper of a country and the average citation per paper of the world (see also [48]). The *RCI* of the country *c* in a given year is defined as $RCI_{c}=\frac{C_{c}}{P_{c}}\times \frac{P_{w}}{C_{w}}$ where *C*_*c*_ and *C*_*w*_ are the total number of citations of the country and of the world, and *P*_*c*_ and *P*_*w*_ the publications of the country and of the world. The total number of citations is the sum of the country self-citations (*S*_*c*_) and the external citation (*X*_*c*_); when the world is considered *C*_*w*_ = *S*_*w*_, since obviously *X*_*w*_ = 0. If a Relative Self-citation Impact is defined as $RSI_{c}=\frac{S_{c}}{P_{c}}\times \frac{P_{w}}{S_{w}}$, the inwardness indicator can be expressed as

$$I_{c}=\frac{RSI_{c}}{RCI_{c}}=(\frac{\frac{S_{c}}{P_{c}}}{\frac{S_{w}}{P_{w}}})\times (\frac{\frac{C_{w}}{P_{w}}}{\frac{C_{c}}{P_{c}}})=\frac{S_{c}}{C_{c}}$$ (2)

Note that the inwardness indicator is normalized for the size of the country in terms of publications.

From a conceptual point of view, the inwardness of a country is an indicator of how much the knowledge produced in the form of scientific publications in a given year in a country flows, through citations, into the knowledge produced in that country in the following years [49–51]. Indeed, 1 − *I*_*c*_ indicates how much of the knowledge produced in a year in a country flows, through citations, into the knowledge (publications) produced by other countries [52, 53]. A higher level of inwardness suggests that the knowledge produced by a country attracts mainly the interest of the national community. By contrast, a lower level suggests that the research of the country does not remain confined within its own borders but flows also toward the rest of the world. It is important to stress that the inwardness, as such, has not an evaluative connotation. The inwardness is a descriptive measure of the self-referentiality of a country in a certain research area. It serves to provide a quantitative indicator of a phenomenon (the self-referentiality), not to judge it.

As said above, the strategic use of citations, both as author self-citations and as citation clubs, affects the country self-citations and, hence, also the inwardness indicator. The start of a strategic use of citations at the country level should therefore be associated with an *anomalous* rise of the inwardness indicator.

Recall, however, that inwardness is positively affected also by increases of international collaboration. It is therefore necessary to control the trend of the international collaboration before concluding that an inwardness rise is due to strategic behaviors and not to an increase of international collaboration.

## Data

We retrieved the data for calculating the Inwardness indicator from SCIval, an Elsevier’s owned platform powered by Scopus data (https://www.scival.com/home). The data were exported from SCIval on October 16, 2018. They correspond to the last update on Scopus of September 21, 2018. Data were retrieved in compliance with the terms of service of SCIval.

In particular, we exported from SCIval two metrics: (1) Citation Count including self-citations, and (2) Citation Count excluding self-citations. For both metrics, we included articles, reviews, and conference papers, leaving aside other types of publications. The first Citation Count metrics represents the countries’ total number of citations, whereas the countries’ number of self-citations was obtained as the difference between (1) and (2). Note that the SCIval’s definition is binary and non-fractional: a citation can either be a self-citation or not [54]. The weight of a country self-citation remains always 1, irrespective of the number of countries producing the citing or the cited publications: if an Italian publication is cited by another Italian publication, this self-citation will have the same weight as if the same publication was cited by an international Italo-French-Chinese publication.

We retrieved the data for the G10 countries (Belgium-BE, Canada-CA, France-FR, Germany-DE, Italy-IT, Japan-JP, the Netherlands-NL, Sweden-SE, Switzerland-CH, United Kingdom-GB, United States-US). In the years 2000-2016, the output of these countries corresponded to 61.2% of the world output and they collected 95% of world citations. In order to study the spread of the strategic behavior in different research areas, data were exported for all the Scopus fields aggregated, i.e., without any filter for subject area, and for each of the 27 Scopus Main Categories (total number of datasets = 28), for the years 2000-2016 included. In order to account for the effect of international collaboration on the inwardness indicators, we retrieved from SCIval also the Percentage of International Collaboration metric for the target countries. The percentage of international collaboration for a country in a given year is defined as the share of publications of the country coauthored by at least one different country. The graphs were implemented in R by using the package “ggplot2” [55].

## Results

**Fig 1:**
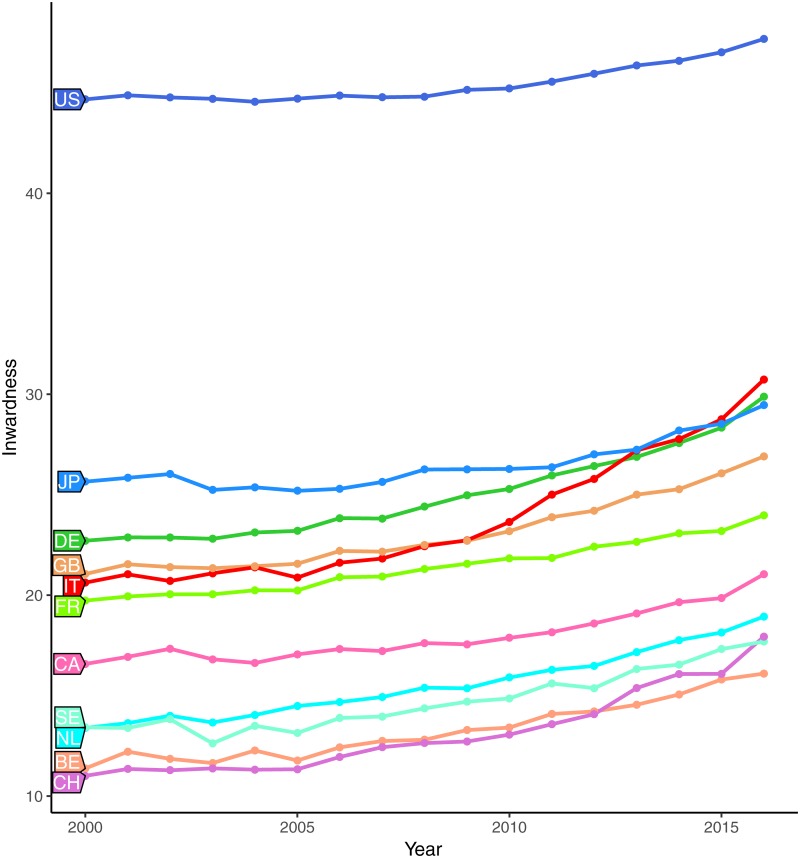
Inwardness for G10 countries (2000-2016). Source: elaboration on SCIval data.

Fig 1 shows the trend of the inwardness over time for the eleven target countries (all Scopus fields aggregated). All countries share a rather similar profile with apparent differences in the absolute value. The ranking is partially explained by the size of the scientific production of the countries. Countries with a large scientific output, such as the Unites States, naturally attract more citations from their own production, simply because they have more citing and citable articles than smaller countries such as Belgium. For all the countries under analysis, not only the inwardness increases slowly and regularly over time, but the yearly ranks of countries according to their inwardness are remarkably stable.

In this landscape, Italy stands out as a notable exception. In 2000, at the beginning of the period, Italy has an inwardness of 20.62% and ranks sixth, just behind UK. In 2016, at the end of the period, Italy ranks second, with an inwardness of 30.73%. Note that, until 2009, Italy’s inwardness grows parallel to those of comparable countries (UK, Germany, France). However, around 2010, the Italian trend shows a sudden acceleration. In the following six years, Italy overcomes UK, Germany, and Japan, becoming the first European country and the second one in the G10 group.

Table 1 shows the variations (deltas) of the inwardness for each country, for the whole period and by considering two sub-periods, 2008-2000 and 2016-2008. Note that in the first period, Italy’s increase is in line with other countries, while in the second period (2008-2016), Italy’s exhibits the largest inwardness delta: 8.29 p.p., more than 4 p.p. above the G10 average and almost 3 p.p. above Germany. As a result, Italy is by far the country with the highest inwardness delta also in the whole period 2000-2016 (10.11 p.p. vs 5.22 of the G10 average).

**Table 1:** Inwardness delta. Delta is calculated as simple difference (p.p.) between the inwardness in the last and the first year of the period.

Country	Δ_1_ (2000-2008)	Δ_2_ (2008-2016)	Δ_*tot*_ (2000-2016)
Belgium	1.42	3.29	4.72
Canada	1.04	3.43	4.46
France	1.57	2.68	4.25
Germany	1.69	5.47	7.17
Italy	1.82	8.29	10.11
Japan	0.6	3.2	3.81
Netherlands	2	3.54	5.54
Sweden	0.94	3.32	4.27
Switzerland	0.94	3.32	4.27
United Kingdom	1.45	4.4	5.85
United States	0.14	2.87	3.01
*Mean G10 countries*	1.24	3.98	5.22

**Fig 2:**
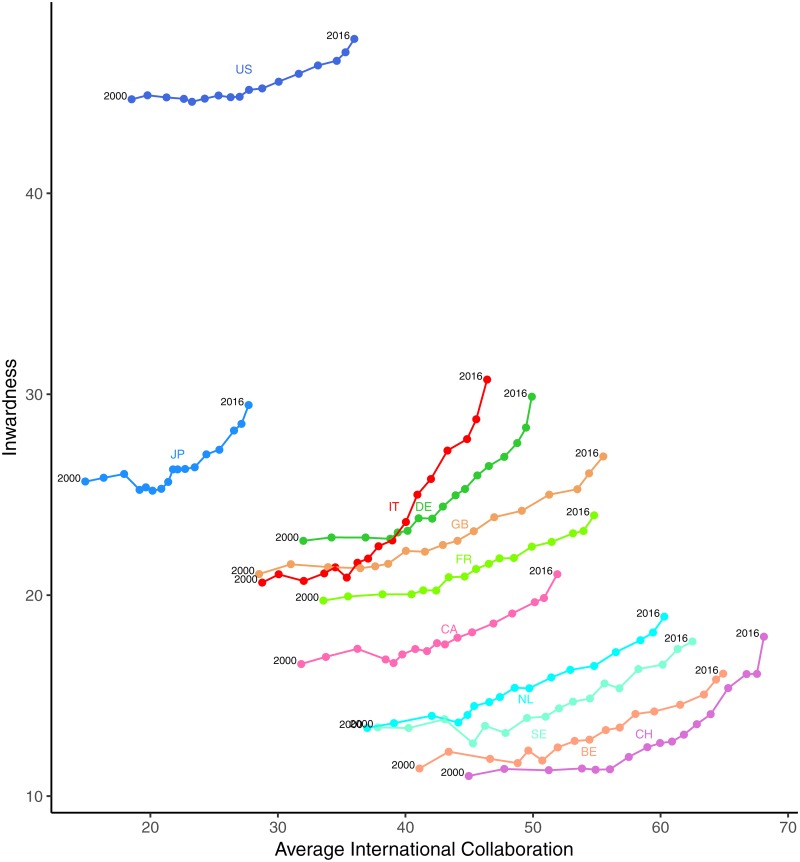
Inwardness versus average international collaboration for the G10 countries. The average international collaboration is the 3-year moving average calculated starting from the considered year. The international collaboration is defined as the share of publications of a country coauthored by at least a coauthor of a different country. Source: elaboration from SCIval data.

However, as already said, inwardness is affected by the amount of International Collaboration of a country. In order to allow for this effect, in Fig 2, inwardness is plotted against the average international collaboration score of each country. More precisely, inwardness at year Y is plotted against the three-years moving average value of international collaboration calculated starting from year Y. In fact inwardness at year Y depends also on citations coming from publications appeared in the following years [56].

The data shows indeed a positive relation between the two variables: for all the countries, inwardness grows with the average international collaboration. The plot shows a peculiar trajectory for Italy. Although for most years Italy ranks last in Europe for international collaboration (x-axis), nevertheless, at the end of the period, it is the first European country for inwardness (y-axis). Before 2010, Italy is close to and moves together with a group of three European countries, namely Germany, UK, and France. Starting from 2010, Italy departs from the group along a steep trajectory, to eventually become the European country *with the lowest international collaboration and the highest inwardness*.

**Fig 3:**
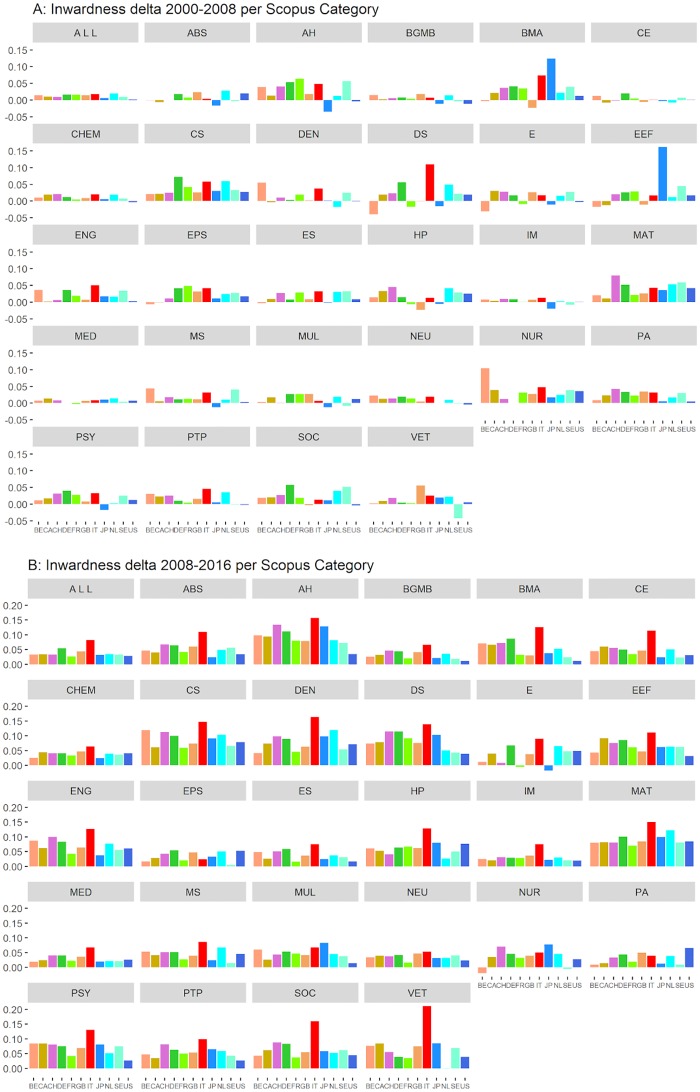
Inwardness delta in Scopus Main Categories in the periods 2000-2008 (A) and 2008-2016 (B). ABS = Agricultural and Biological Sciences, AH = Arts and Humanities, BGMB = Biochemistry, Genetics and Molecular Biology, BMA = Business, Management and Accounting, CE = Chemical Engineering, CHEM = Chemistry, CS = Computer Science, DEN = Dentistry, DS = Decision Sciences, E = Energy, EEF = Economics, Econometrics and Finance, ENG = Engineering, EPS = Earth and Planetary Sciences, ES = Environmental Science, HP = Health Professions, IM = Immunology and Microbiology, MAT = Mathematics, MED = Medicine, MS = Materials Science, MUL = Multidisciplinary, NEU = Neuroscience, NUR = Nursing, PA = Physics and Astronomy, PSY = Psychology, PTP = Pharmacology, Toxicology and Pharmaceutics, SOC = Social Sciences, VET = Veterinary. Source: elaboration from SCIval data.

Until now, we focused on the aggregated output of the target countries, without considering the different research areas (Scopus Main Categories). In order to investigate whether and how inwardness changes across research areas, we calculated the inwardness time series for each of the 27 Scopus Main Categories. The time series, as well as the scatterplots of the inwardness against the international collaboration, are fully provided in the Supplementary Materials. For reasons of space, these data are summarized in Fig 3, where the variation of the inwardness indicator in the periods 2000-2008 (A) and 2008-2016 (B) is displayed for each of the 27 Scopus Categories. Italy shows a remarkable difference between the two periods. In the first one (Fig 3A), before the university reform, Italy is in line with the other G10 countries in most of the research fields. In the second period, after the reform (Fig 3B), Italy stands out with the highest inwardness increase in 23 out of 27 fields. The only exceptions are earth and planetary sciences (EPS), multidisciplinary (MUL), nursing (NUR), and physics and astronomy (PA).

As we show in the Supplementary Information (S1 Fig, 1-27), the inwardness increase is *not* matched by a parallel increase of the international collaboration at the field level. In particular, at the end of the period, Italy is the European country with the lowest level of international collaboration and the highest value of inwardness in the following Scopus Categories (11 on 27): agricultural and biological sciences (ABS), biochemistry, genetics and molecular biology (BGMB), chemical engineering (CE), economics, econometrics and finance (EEF), earth and planetary sciences (EPS), environmental science (ES), immunology and microbiology (IM), pharmacology, toxicology and pharmaceutics (PTP), veterinary (VET). In other 9 Categories, Italy is first for inwardness but not the lowest for international collaboration: business, management and accounting (BMA), computer science (CS), dentistry (DEN), decision sciences (DS), engineering (ENG), health professions (HP), mathematics (MAT), materials science (MS), psychology (PSY). Note that the Italian production in the arts and humanities (AH) and social sciences (SOC) is only partially covered by Scopus as a large part is published in books and in the national language. Therefore, the results about these scholarly areas should be taken with great caution [57].

## Discussion

As seen from Fig 1 and Table 1, Italy shows a different trend compared to the other G10 countries. The comparative analysis of the inwardness indicator showed that Italian research grew in insularity in the years after the adoption of the new rules of evaluation. While the level of international collaboration remained stable and comparatively low, the research produced in the country tended to be increasingly cited by papers authored by at least an Italian scholar.

The anomalous trend of the inwardness indicator detected at the macro level can be explained by a generalized change in micro-behaviours of Italian researchers induced by the introduction of bibliometric thresholds in the national regulations for recruitment and career advancement. Indeed, in 2011 research and careers evaluation were revolutionized by the introduction of quantitative criteria in which citations played a central role. In particular, citations started being rewarded in the recruiting and habilitation mechanisms, *regardless of their source*. This created an incentive to inflate those citation scores by means of strategic behaviors, such as opportunistic self-citations and the creation of citation clubs.

A possible objection to the above explanation is that, in order to postulate individual and collective behaviors, the collection of evidence at the micro level is an indispensable step. According to this objection, unless you draw on co-authorship networks, you should avoid talking about citations clubs, citations cartels, and citation gaming. Evidence, for instance, could be searched by checking the existence of groups of researchers frequently exchanging citations, that are not directly co-authors but at a relatively small distance in a co-authorship network. Without this kind of micro level analysis, one could just record the increase of inwardness as a response to the reformation of the Italian reward system, but should not hazard an explanation at the micro level.

As a matter of fact, a simple argument, based on set theory, shows that the above objection is unduly conservative. The set *C* of the country self-citations is the union of two sets (*C* = *A* ∪ *B*): the self-citations *A* generated by country-based researchers as a *normal byproduct* of the research activity and the self-citations *B* resulting from *strategic activities*, including both opportunistic self-citation and country-based citation clubs. Put in other words, the set *A* is the “physiological” quota of country self-citations, whereas *B* is the “pathological” quota. An increase of the inwardness indicator is, by definition, an increase of the cardinality of the set *C* of the country self-citations. There are two *possible explanations* for that increase: (i) the cardinality of *A* has increased, i.e. the physiological quota *A* of country self-citations has increased; or (ii) the cardinality of *B* has increased, i.e. the pathological quota *B* of country self-citations has increased.

Two explanations for an increase of cardinality of physiological quota *A* could be advanced. According to the first one, *internationalization*, the increase may be due to a sudden rise, after 2009, of the amount of international collaborations of Italian scholars. In fact, we have already observed that, other things left unchanged, an increase of international collaboration positively affects the inwardness indicator. However, Fig 2 rules out this explanation. No peculiar increase in the Italian international collaboration can be spotted.

The second explanation, *specialization*, is a narrowing of the scientific focus of Italian researchers, i.e. a dynamic of scientific specialization leading to the growth of author self-citations [32]. The idea is that focusing on narrower topics results in a contraction of the scientific community of reference. Thus, the number of citable papers would diminish and the chances for author self-citation would correspondingly increase, generating also the growth of the country self-citations. Although we do not have direct evidence falsifying the specialization hypothesis, nonetheless, this explanation appears largely implausible. Indeed, it implies that Italian researchers in all fields suddenly narrowed their focus to topics mainly investigated in the national community. This sudden change would be not only peculiar of Italy, but also so strong as to make the Italian inwardness diverge from those of the other G10 countries. Notably, Fig 3 shows that the Italian post-2008 acceleration is visible in most of the research areas. Not only the change has been widespread, regarding most research fields, but in some of them, such as engineering (ENG), mathematics (MAT) or veterinary (VET), the increase reached outstanding proportions. In any case, it would still be necessary to explain why a physiological specialization occurred only in Italy and at the same time as the adoption of new rules for evaluation.

Summing up, we have no plausible reasons in favor of a notable change in the physiological quota *A* of country self-citations, sufficient to explain the anomalous boost of inwardness with respect to the other G10 countries. Recalling that *C* = *A* ∪ *B*, the only alternative explanation of the change in the cardinality of *C* is a notable expansion of the pathological set *B* of country self-citations, i.e., an increase of author self-citations and an increase of citations exchanged within citation-clubs formed by Italian scholars, aimed at boosting bibliometric indicators set by ANVUR.

The slight discrepancy between the starting year of the inwardness acceleration and the launch of bibliometric evaluation system, with the former occurring slightly earlier than the latter, is easily explained by the “backward effect” typical of citation measures. Any change in the citation habits taking place in a given year produces a backward effect on the citation scores of the previous years because researchers cite previously published papers, so that the change reverberates also on the citation scores of the past production. Citations received by the most recent articles have a more lasting effect in the calculations of forthcoming indicators. It is therefore more convenient to self-cite one’s own recent production rather than the remote one. Hence, a strategic reaction to rules introduced in year 2011 is expected to produce an inwardness acceleration that starts a few years before, just as observed for Italy.

## Conclusions

In this paper, we contributed to the empirical study of the constitutive effects that indicator-based research evaluation systems have on the behavior of the evaluated researchers. By focusing on the Italian case, we investigated how the Italian scientific community responded, *at the national level*, to the introduction of a research evaluation system, in which bibliometric indicators play a crucial role. Our results show that the behavior of Italian researchers has indeed changed after the introduction of the evaluation system following the 2010 university reform. Such a change is visible *at a national scale in most of the scientific fields*. The comparative analysis of the inwardness indicator showed that Italian research grew in insularity in the years after the adoption of the new rules of evaluation. While the level of international collaboration remained stable and comparatively low, the research produced in the country tended to be increasingly cited by papers authored by at least an Italian scholar.

We explained this as the result of the pervasively adoption of strategic citation behaviors within the Italian scientific community. Even if they escape a direct observation, we argue that such behaviors are the *most likely explanation* of the peculiar trend exhibited by the Italian inwardness. This because our indicator was especially designed to be sensible to the effects of both the opportunistic use of author self-citation and the creation of citation clubs.

We believe that three main lessons can be derived from the Italian case. Firstly, our results support the claim that scientists are *quickly responsive* to the system of incentives in which they act [32]. Thus, any policy aiming at introducing or modifying such a system should be designed and implemented very carefully. In particular, considerable attention should be placed on the constitutive effects of bibliometric indicators. They are not neutral measures of performance but actively interact and quickly shape the behavior of the evaluated researchers.

Secondly, our results show that the “responsible use” of metrics would not be enough to prevent the emergence of strategic behaviors. For instance, the Leiden Manifesto recommends the use of a “suite of indicators” instead of a single one as a way to prevent gaming and goal displacement (see the principle number 9 in [13]). The Italian case shows that, even if the researchers are evaluated against multiple indicators, as recommended, strategic behaviors manifest themselves anyway.

Lastly, our results prompt some reflections on the viability of the mixed evaluation systems, in which the indicators are intended for complementing or integrating the expert judgment expressed by the peer review. In fact, the Italian system was designed in principle according to such a mixed approach, both for the research assessment exercises where research products were evaluated by bibliometric indicators or by peer reviewers, and for the ASN where to overcome bibliometric thresholds is but a necessary condition for being admitted to the final evaluation by habilitation committees. Nonetheless, our results show that the mere presence of bibliometric indicators in the evaluative procedures is enough to structurally affect the behavior of the scientists, fostering opportunistic strategies. Therefore, there is the concrete risk that in mixed evaluation systems, the indicator-based component overcomes the peer review-based one. Hence, they *de facto* collapse to indicator-centric approaches. We believe that further research is needed to better understand and fully appreciate the possibility of such a collapse. In the meantime, we suggest that policy makers should exercise the most extreme caution in the use of indicators in science policy contexts.

## Supporting information

S1 Fig 1-27—Inwardness over time (left) and inwardness vs average international collaboration (right) for the G10 countries in each of the Scopus Main Categories. (PDF)

S1 File 1-28—Zipped CSV files for the G10 countries. Files 1-27: data for each Scopus Category; File 28 data for all the Scopus Main Categories aggregated.
(ZIP)
