# Supplementary material for: Citation gaming induced by bibliometric evaluation: A country-level comparative analysis
Source: PLoS One. 2019 Sep 11;14(9):e0221212. doi: 10.1371/journal.pone.0221212 (PMC6739054; doi:10.1371/journal.pone.0221212)
Supplement: S1 Fig — (PDF) [file pone.0221212.s001.pdf]

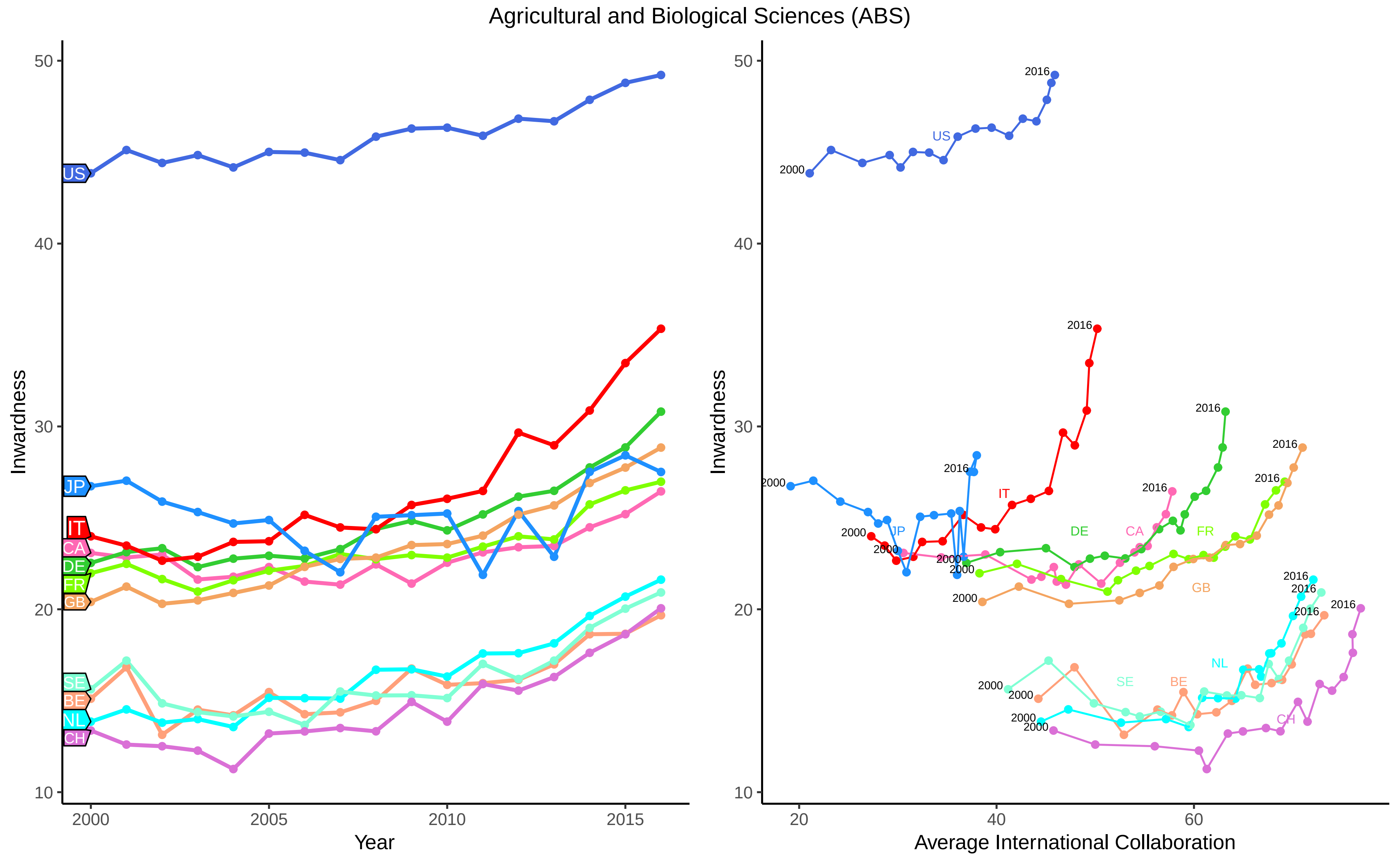

S1 Figure 1. Inwardness over time (left) and inwardness vs average international collaboration (right) for the G10 countries in Agricultural and Biological Sciences (ABS)

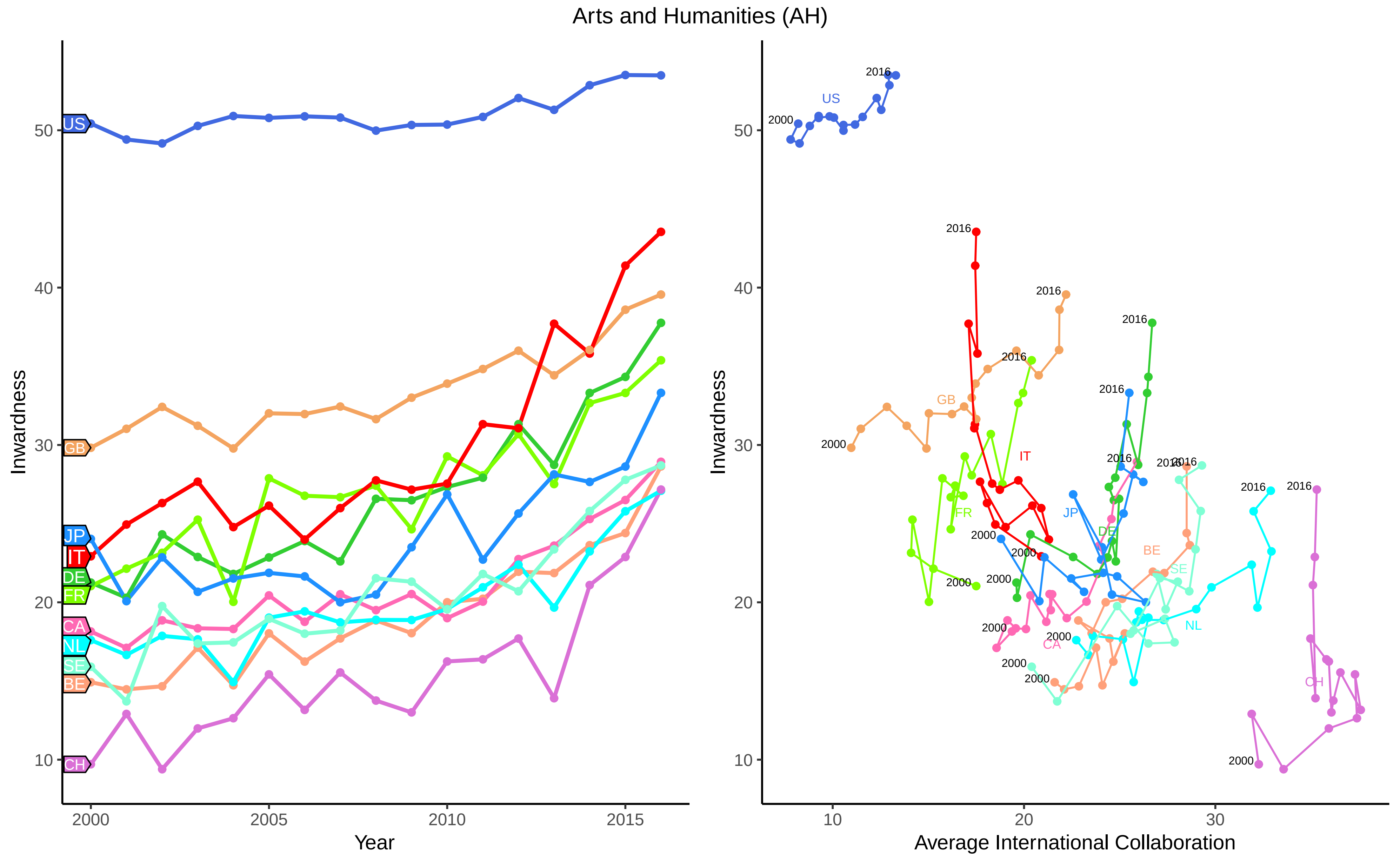

S1 Figure 2. Inwardness over time (left) and inwardness vs average international collaboration (right) for the G10 countries in Arts and Humanities (AH)

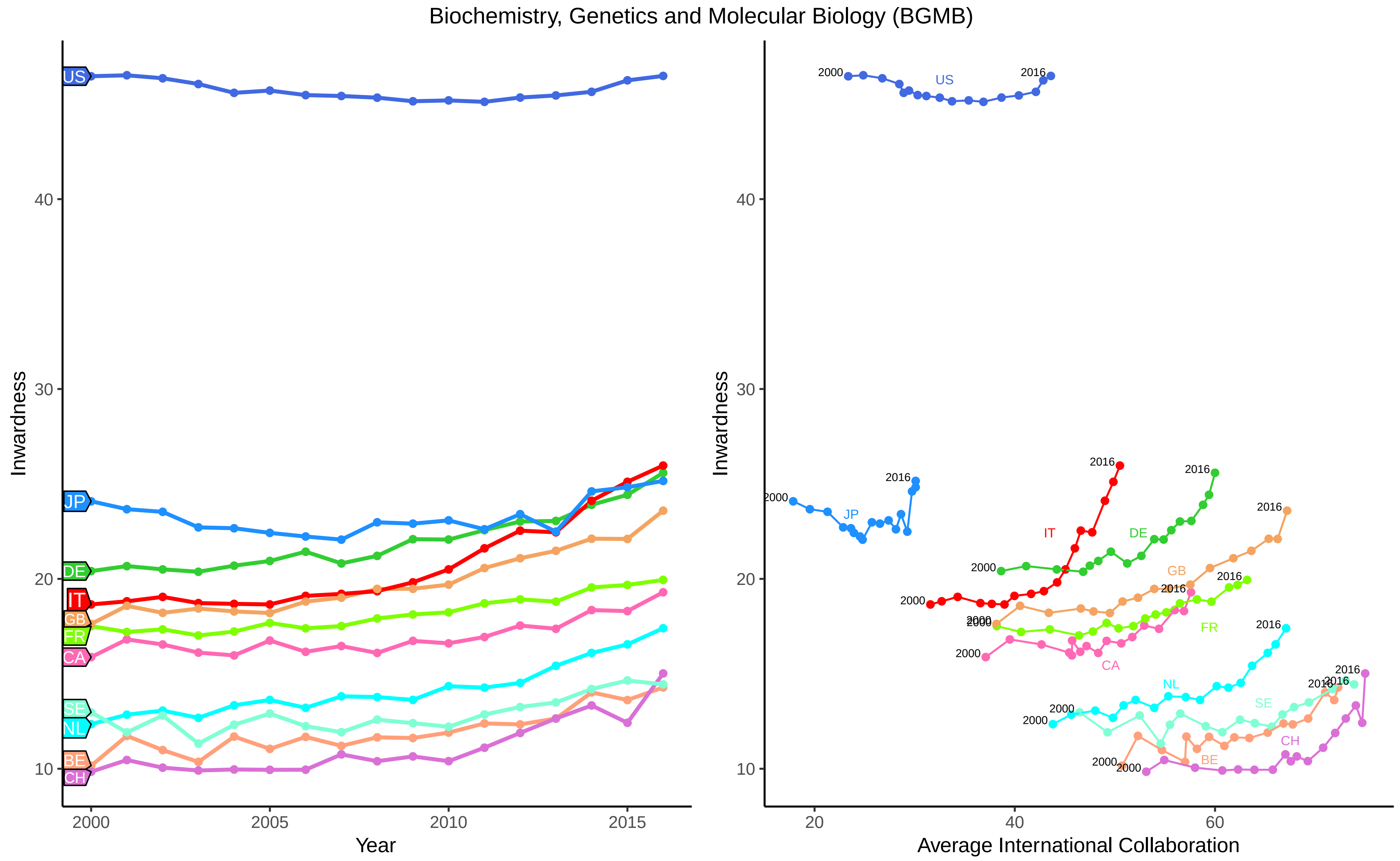

S1 Figure 3. Inwardness over time (left) and inwardness vs average international collaboration (right) for the G10 countries in Biochemistry, Genetics and Molecular Biology (BGMB)

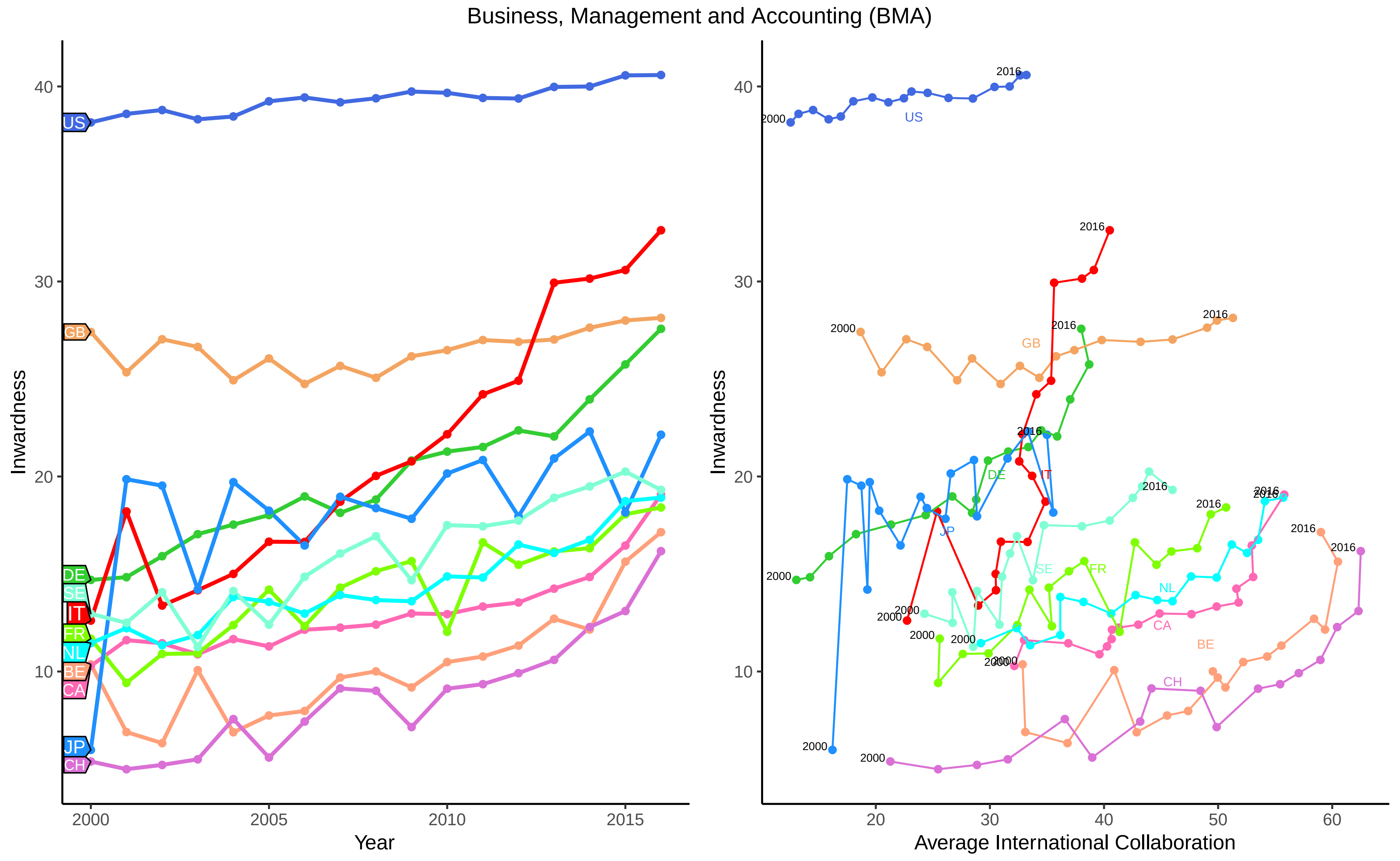

S1 Figure 4. Inwardness over time (left) and inwardness vs average international collaboration (right) for the G10 countries in Business, Management and Accounting (BMA)

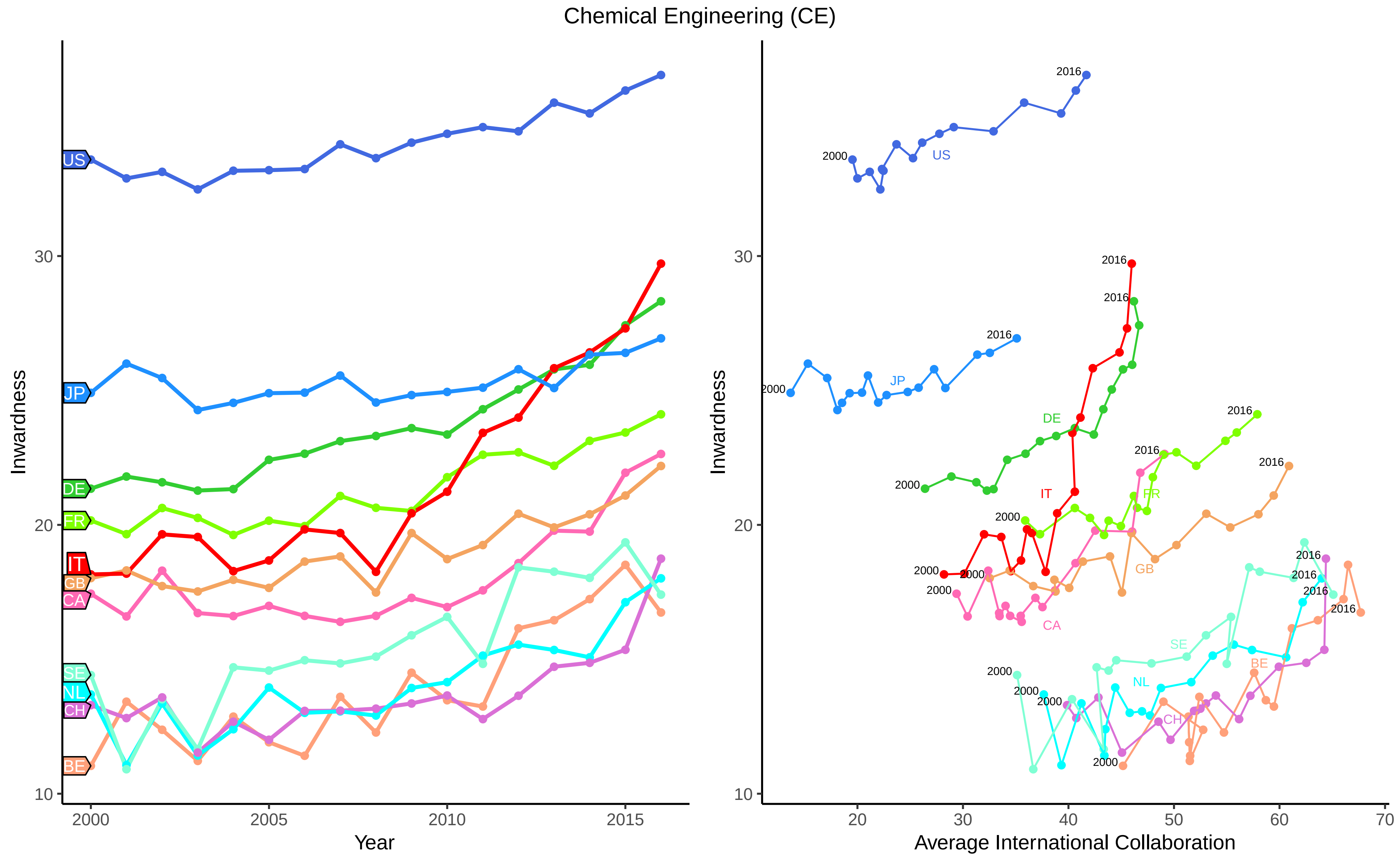

S1 Figure 5. Inwardness over time (left) and inwardness vs average international collaboration (right) for the G10 countries in Chemical Engineering (CE)

Chemistry (CE)

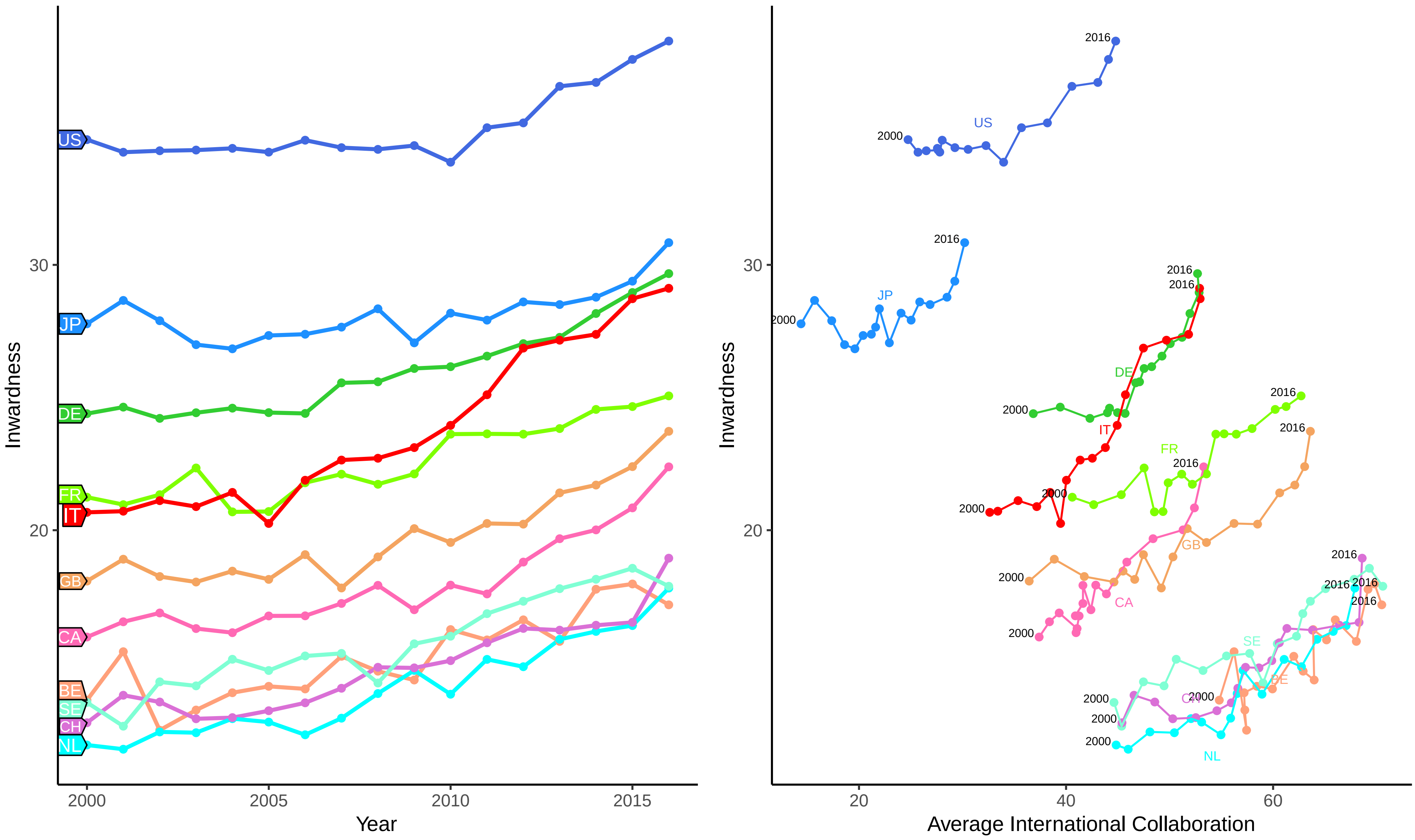

S1 Figure 6. Inwardness over time (left) and inwardness vs average international collaboration (right) for the G10 countries in Chemistry (CE)

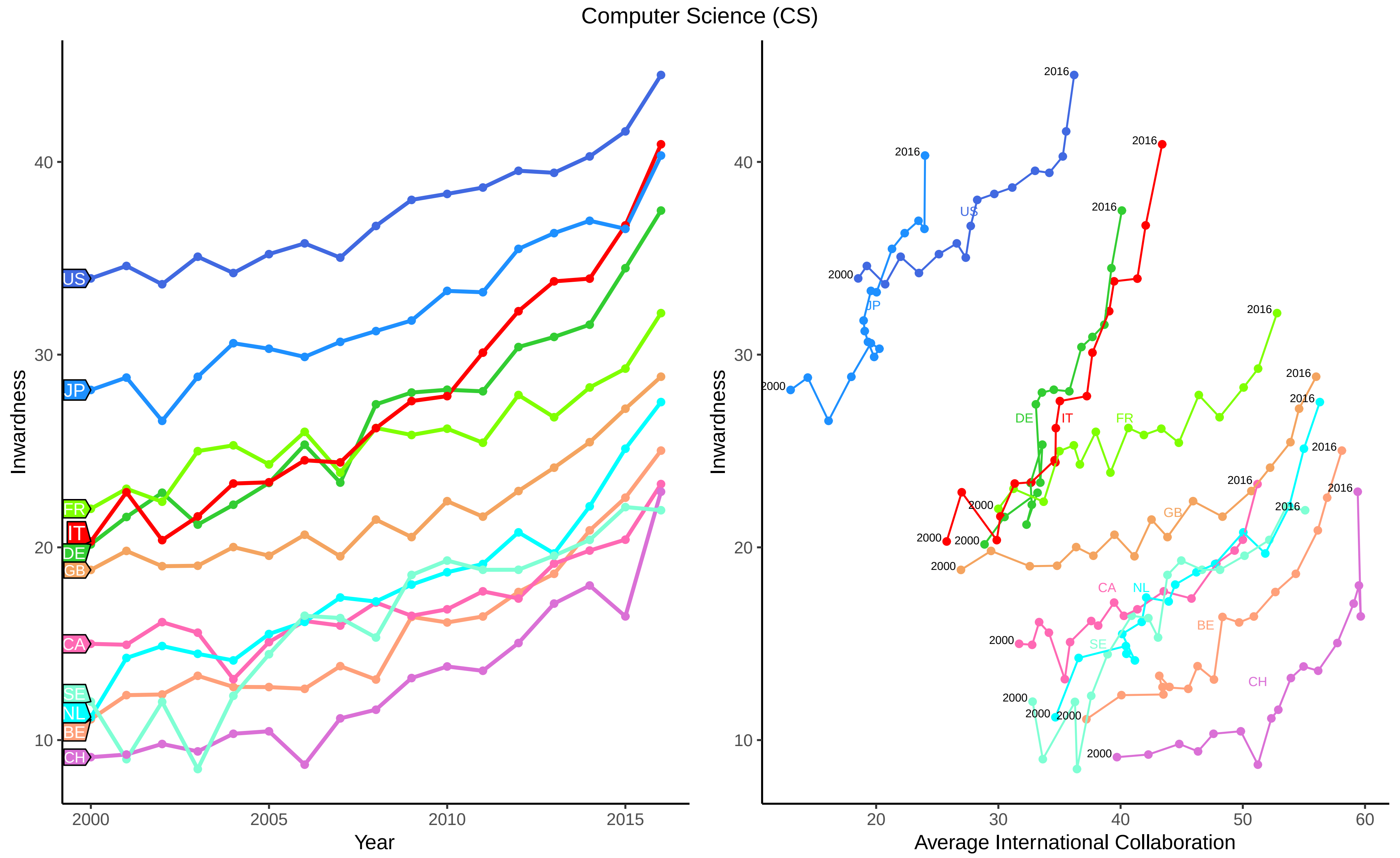

S1 Figure 7. Inwardness over time (left) and inwardness vs average international collaboration (right) for the G10 countries in Computer Science (CS)

Dentistry (DEN)

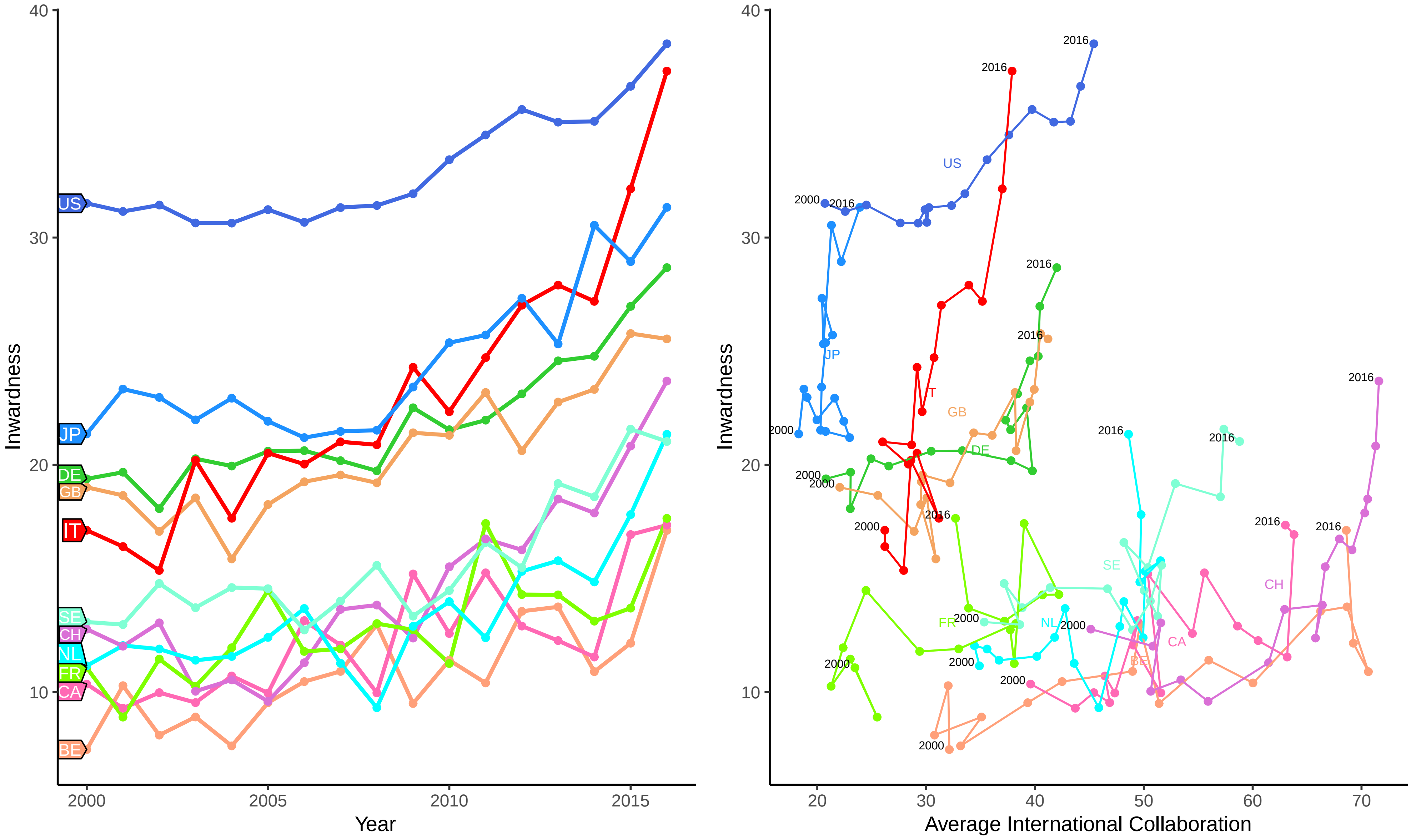

S1 Figure 8. Inwardness over time (left) and inwardness vs average international collaboration (right) for the G10 countries in Dentistry (DEN)

Decision Sciences (DS)

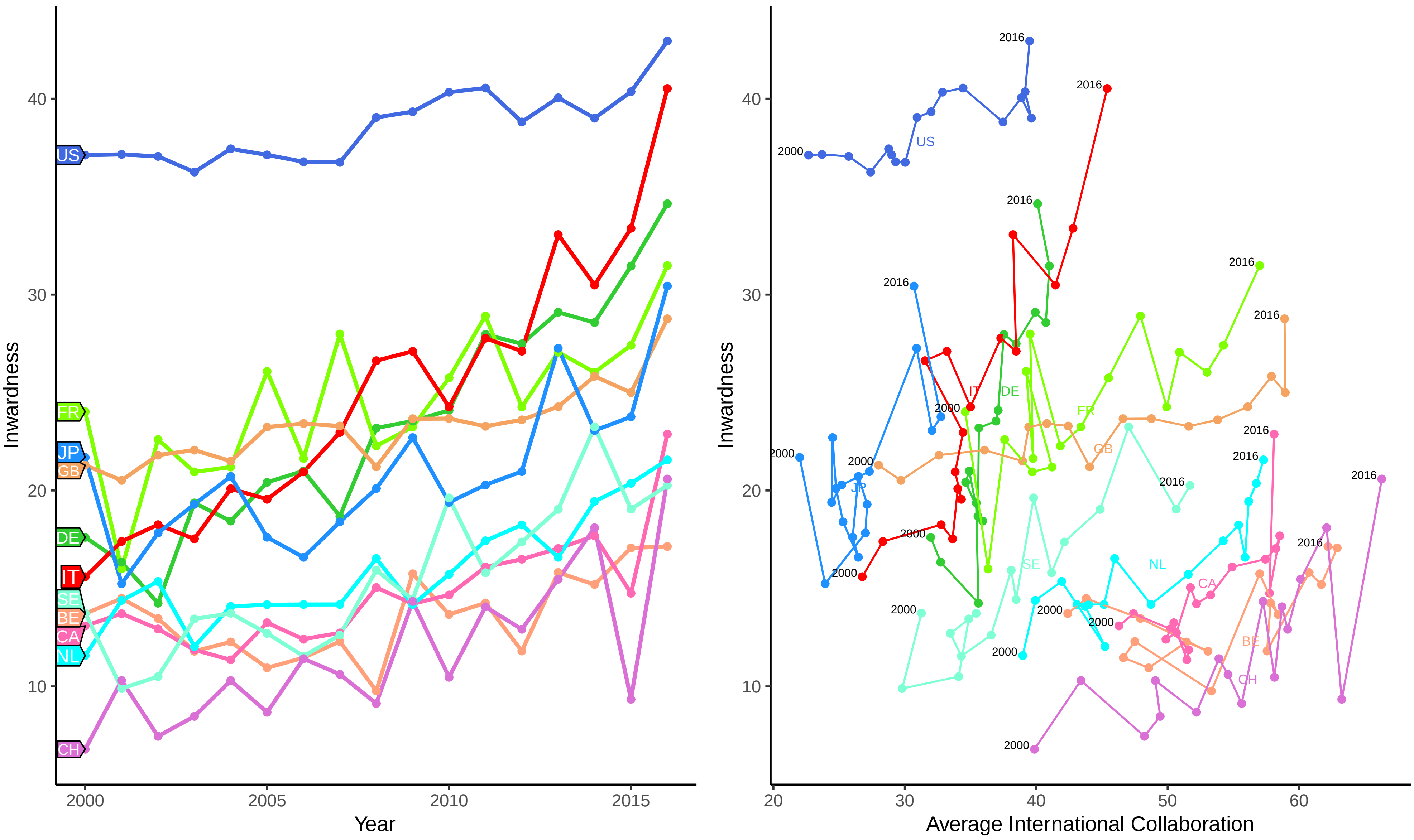

S1 Figure 9. Inwardness over time (left) and inwardness vs average international collaboration (right) for the G10 countries in Decision Sciences (DS)

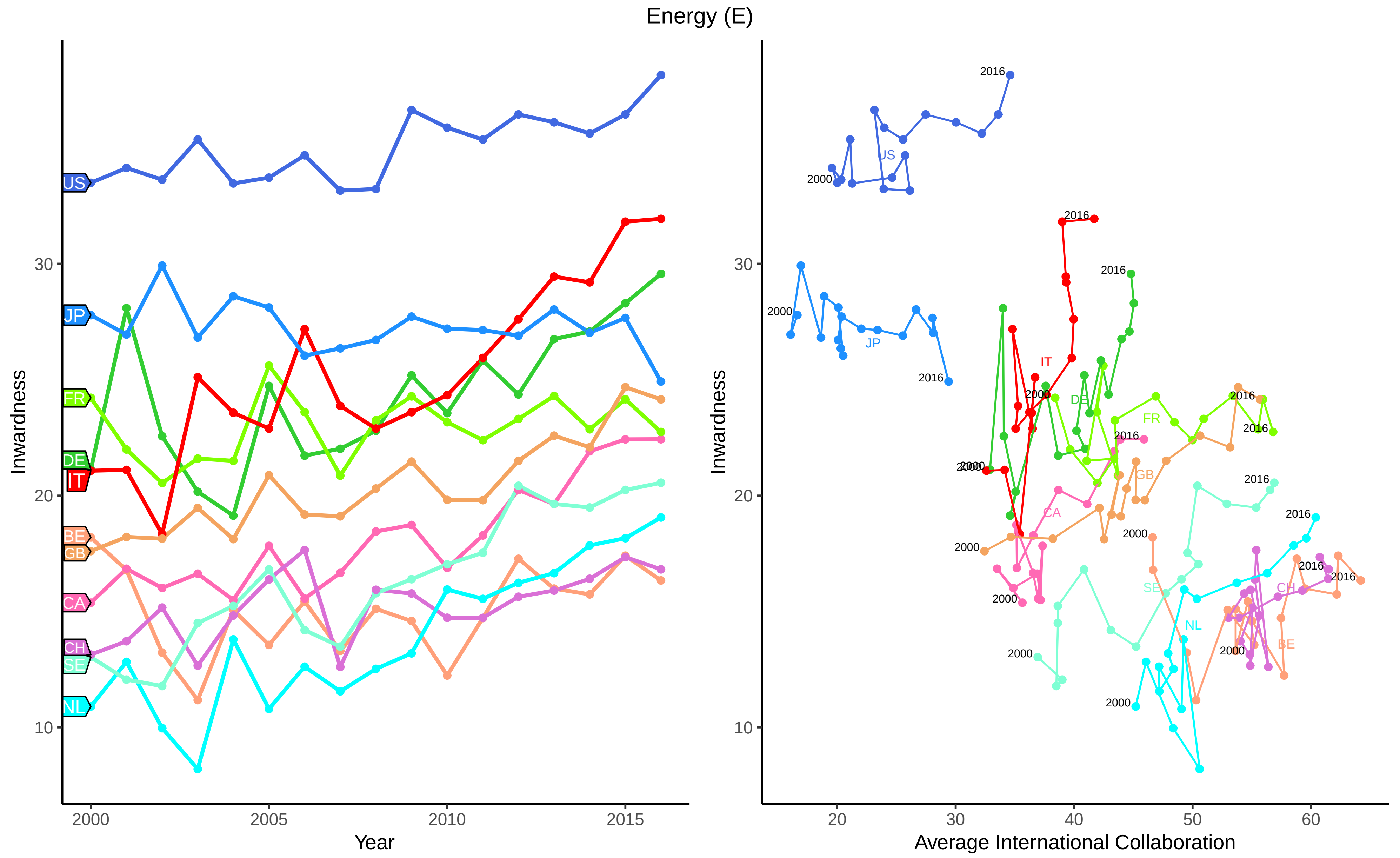

S1 Figure 10. Inwardness over time (left) and inwardness vs average international collaboration (right) for the G10 countries in Energy (E)

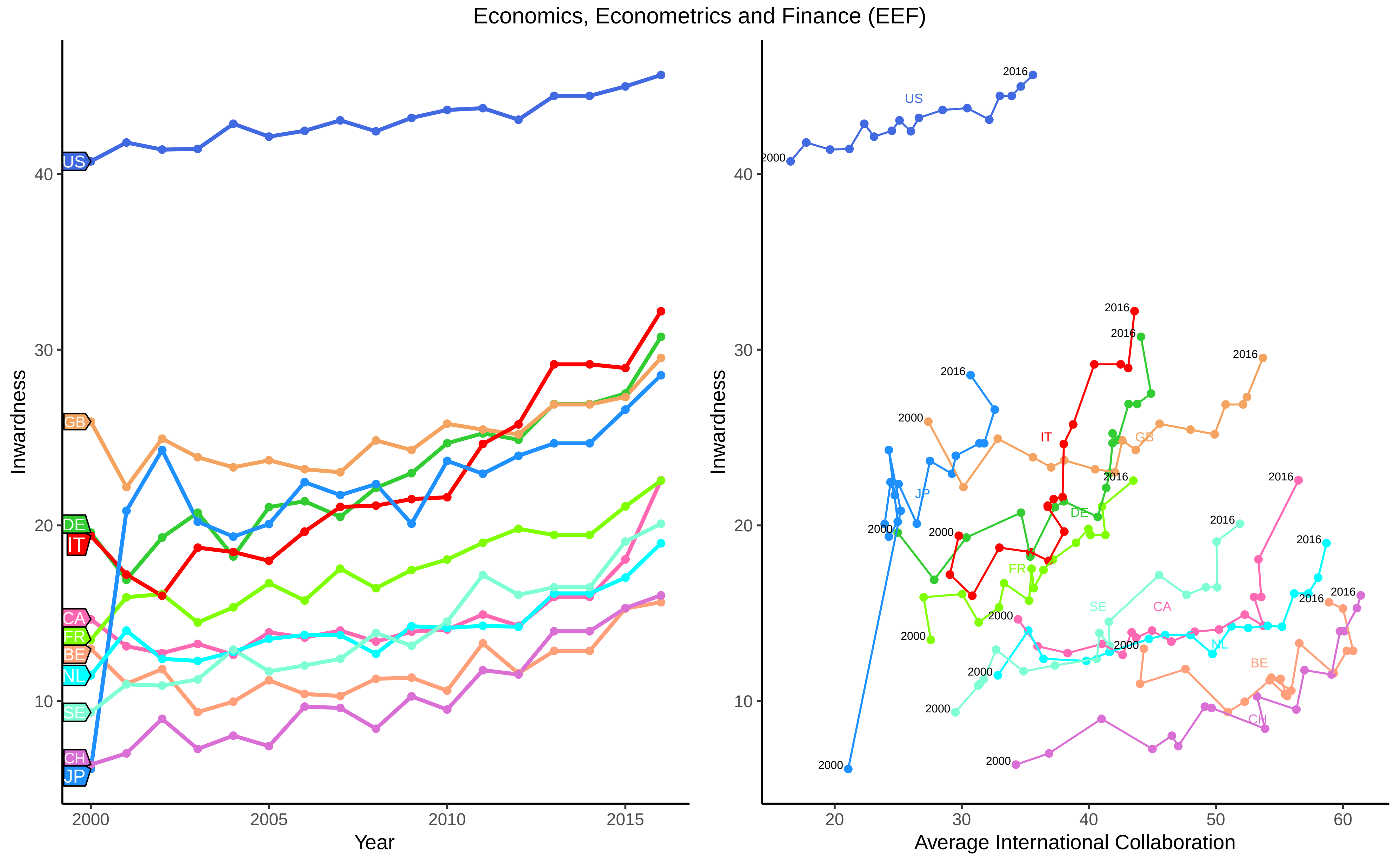

S1 Figure 11. Inwardness over time (left) and inwardness vs average international collaboration (right) for the G10 countries in Economics, Econometrics and Finance (EEF)

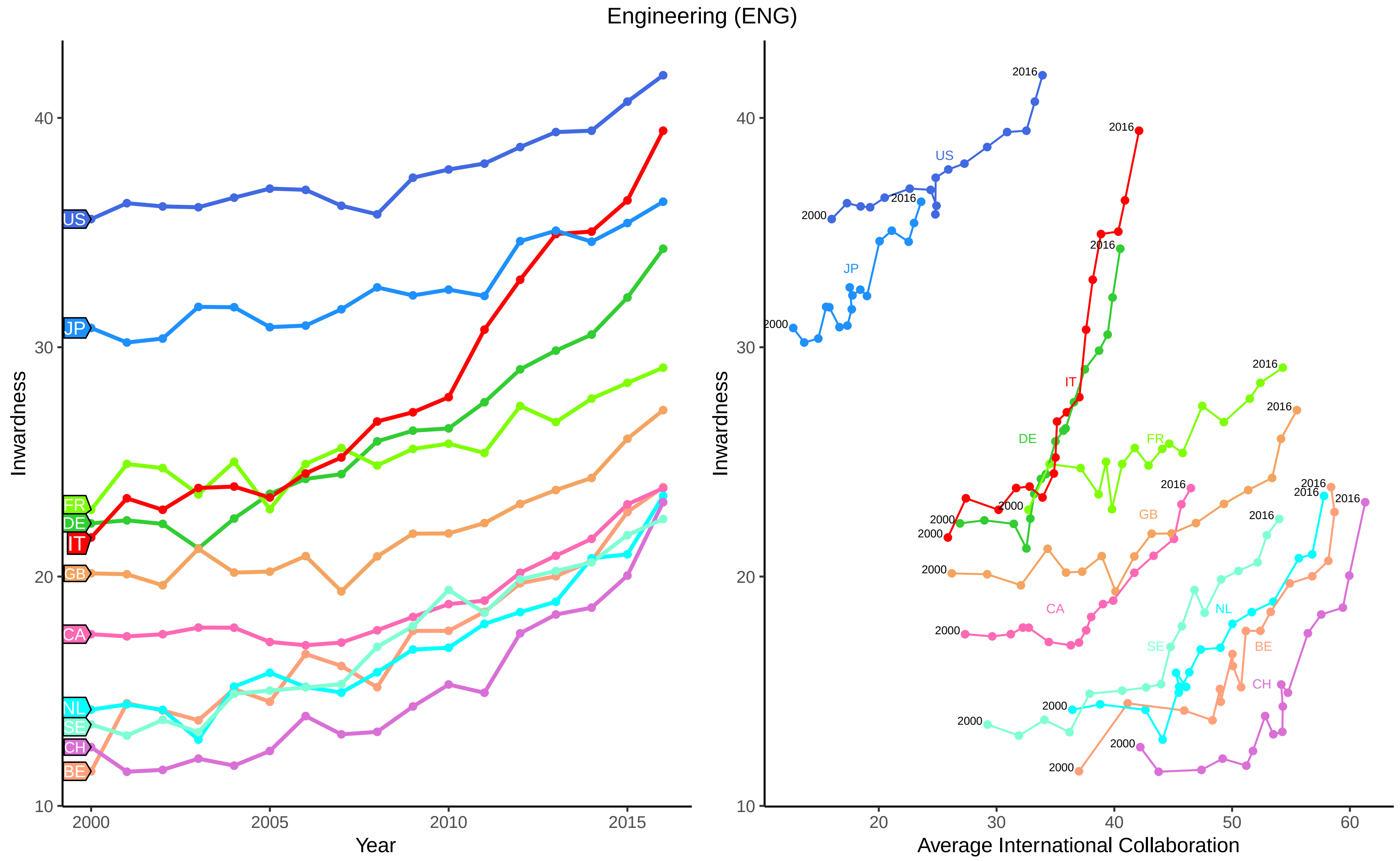

S1 Figure 12. Inwardness over time (left) and inwardness vs average international collaboration (right) for the G10 countries in Engineering (ENG)

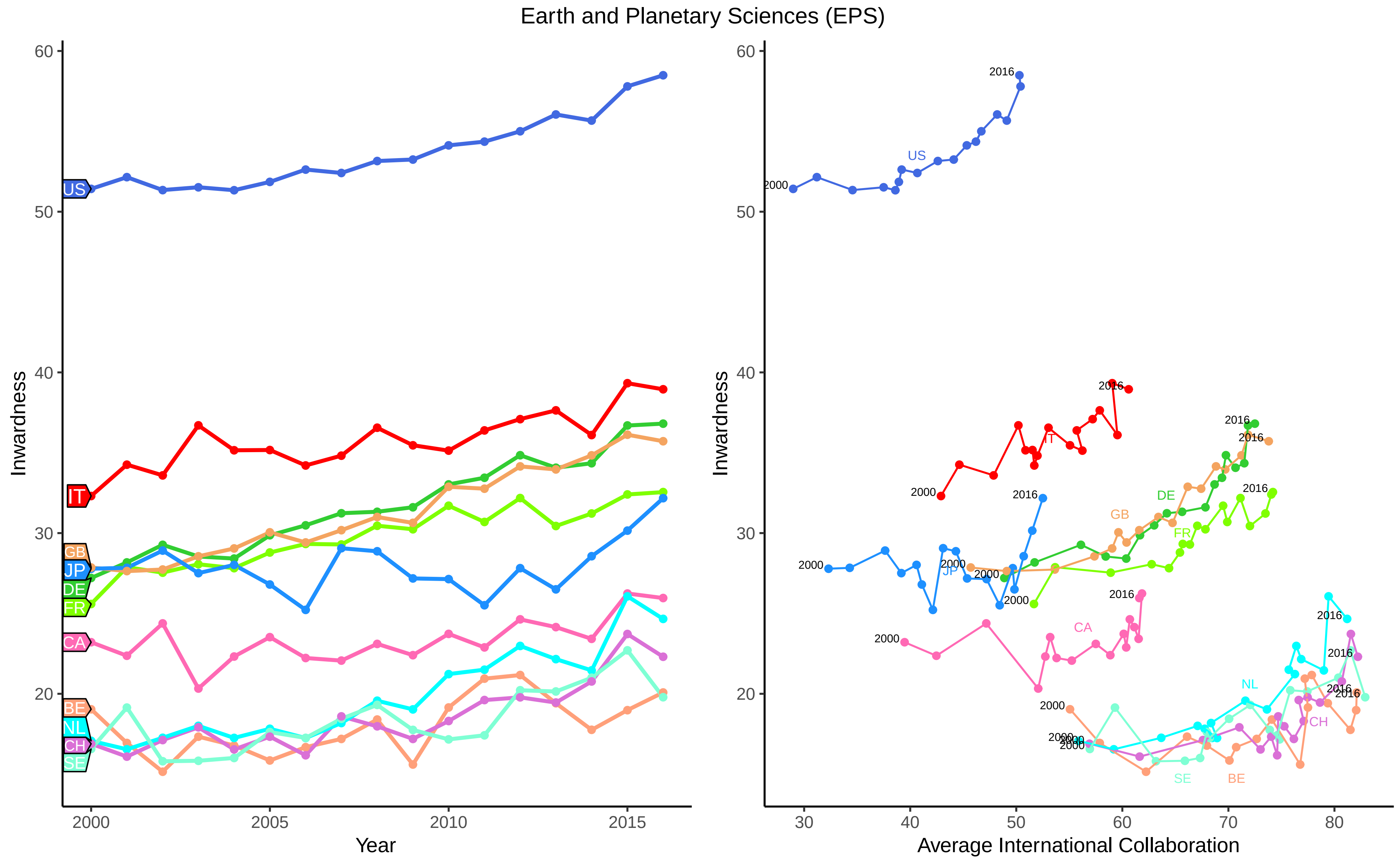

S1 Figure 13. Inwardness over time (left) and inwardness vs average international collaboration (right) for the G10 countries in Earth and Planetary Sciences (EPS)

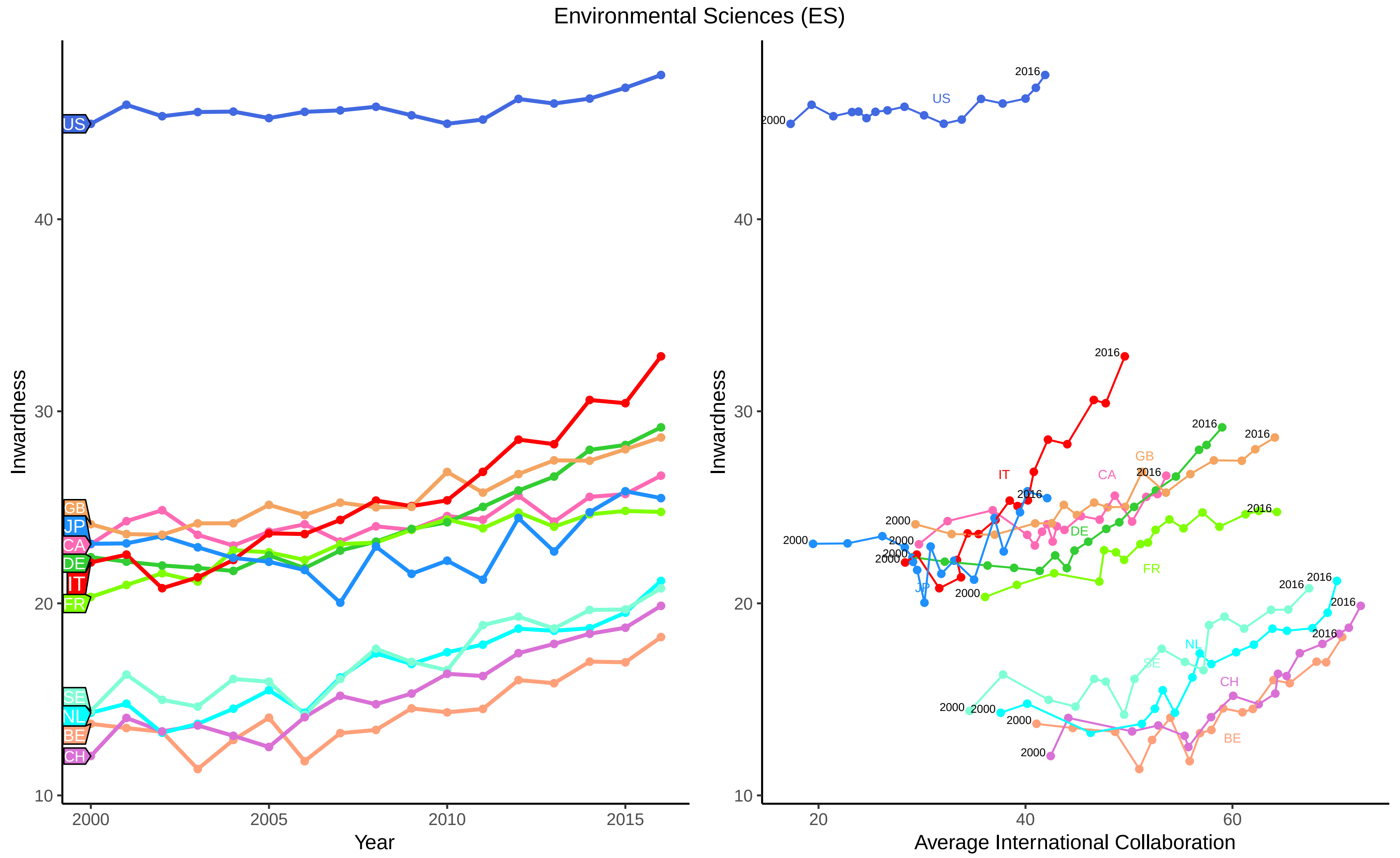

S1 Figure 14. Inwardness over time (left) and inwardness vs average international collaboration (right) for the G10 countries in Environmental Sciences (ES)

Health Professions (HP)

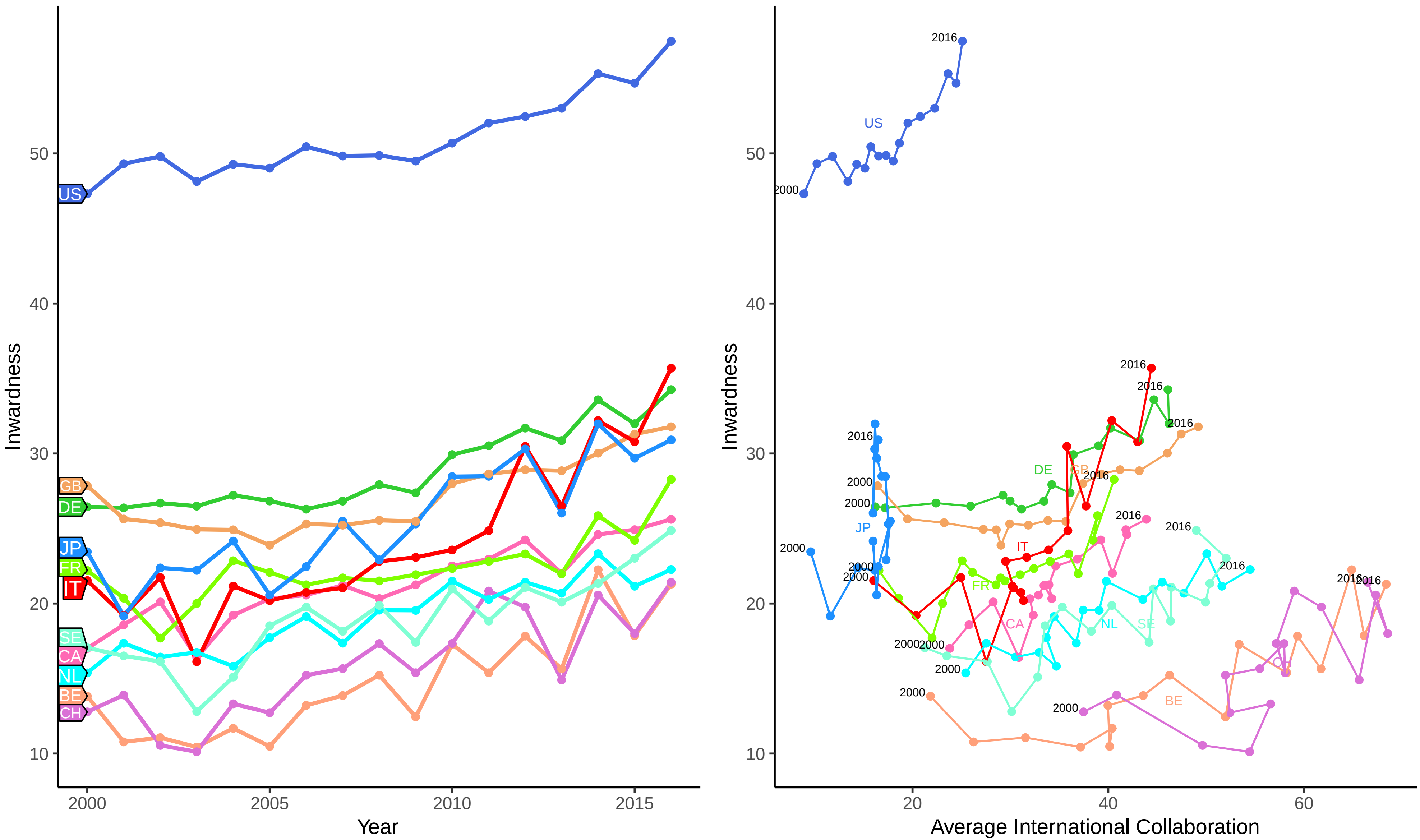

S1 Figure 15. Inwardness over time (left) and inwardness vs average international collaboration (right) for the G10 countries in Health Professions (HP)

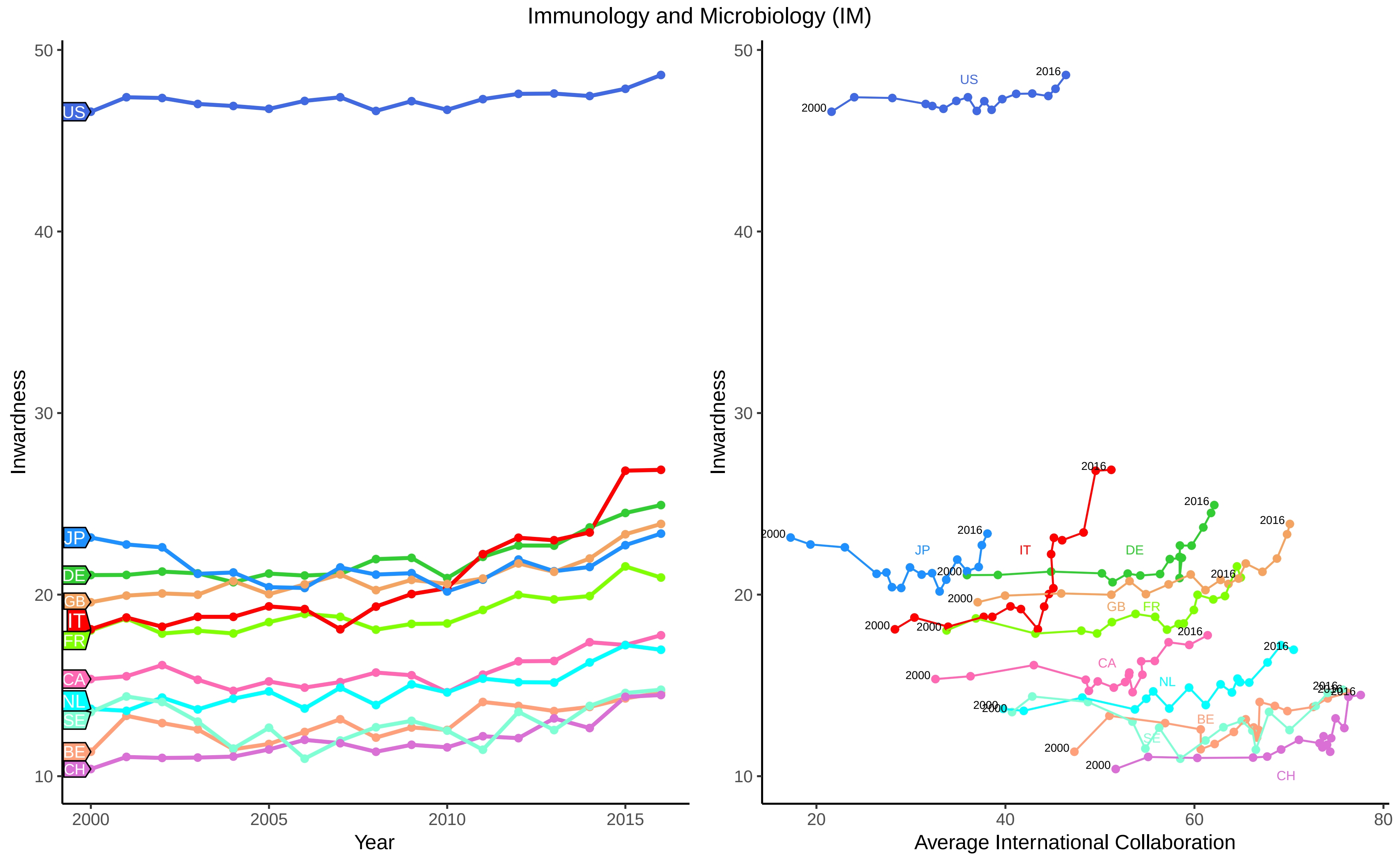

S1 Figure 16. Inwardness over time (left) and inwardness vs average international collaboration (right) for the G10 countries in Immunology and Microbiology (IM)

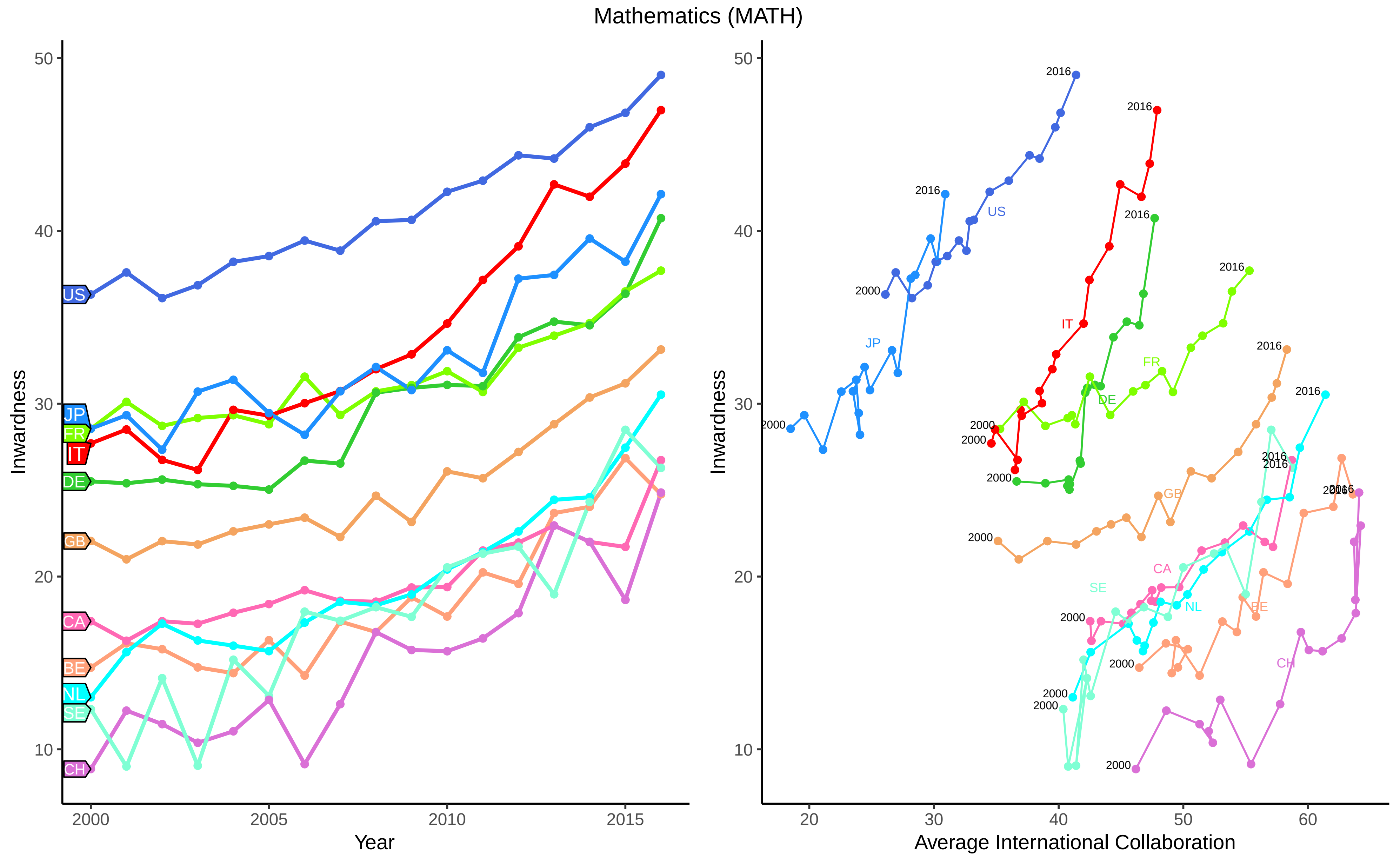

S1 Figure 17. Inwardness over time (left) and inwardness vs average international collaboration (right) for the G10 countries in Mathematics (MAT)

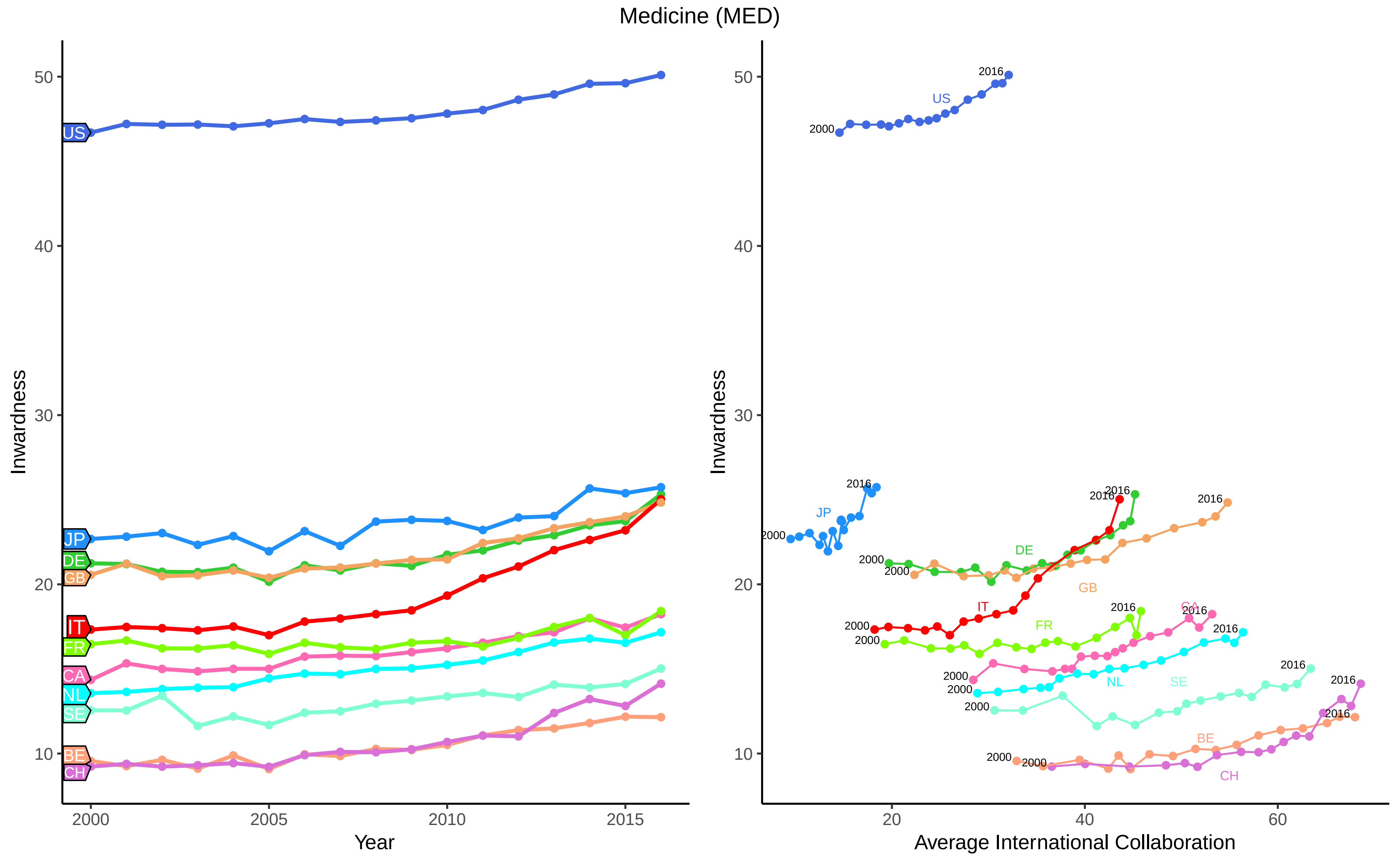

S1 Figure 18. Inwardness over time (left) and inwardness vs average international collaboration (right) for the G10 countries in Medicine (MED)

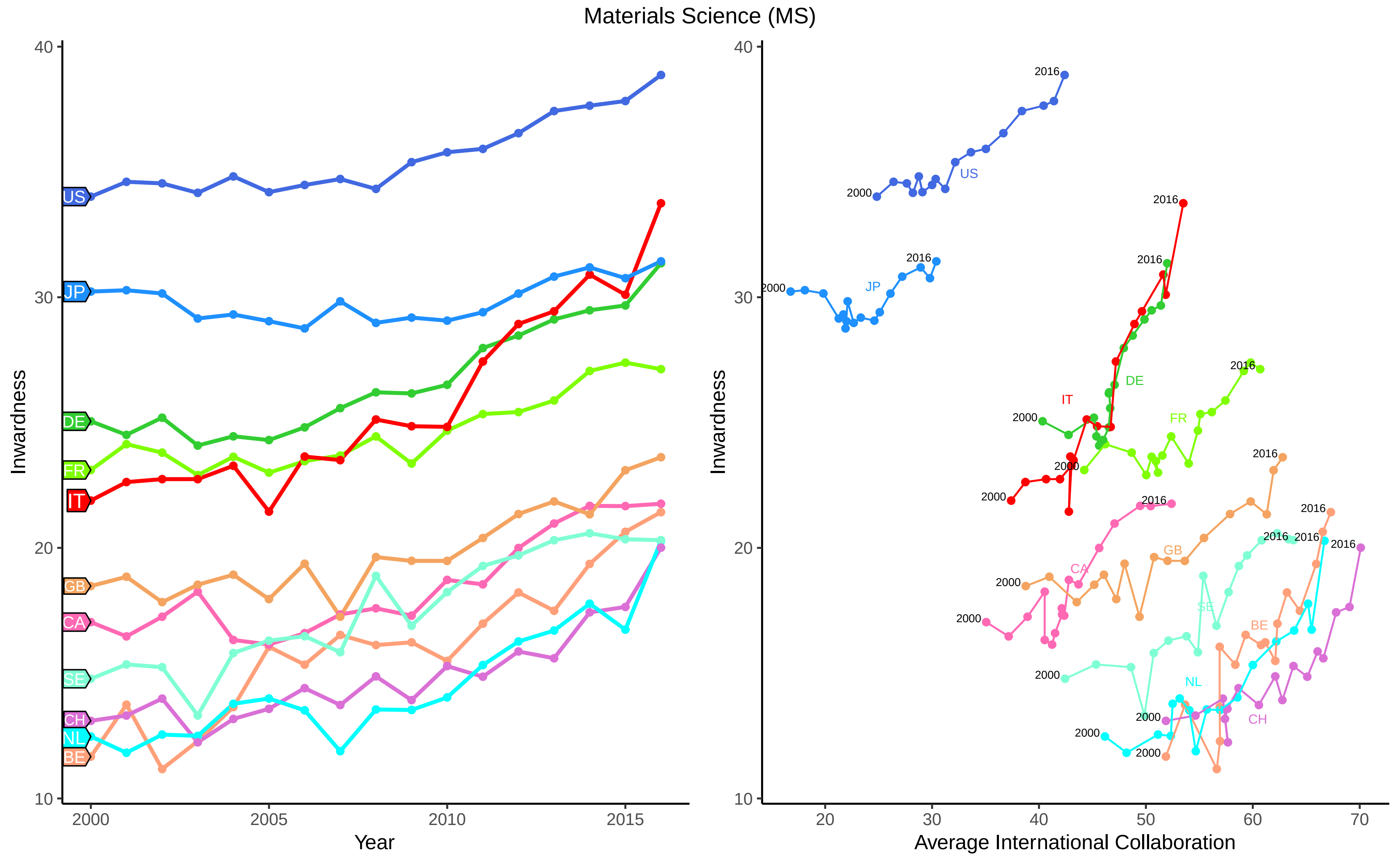

S1 Figure 19. Inwardness over time (left) and inwardness vs average international collaboration (right) for the G10 countries in Materials Science (MS)

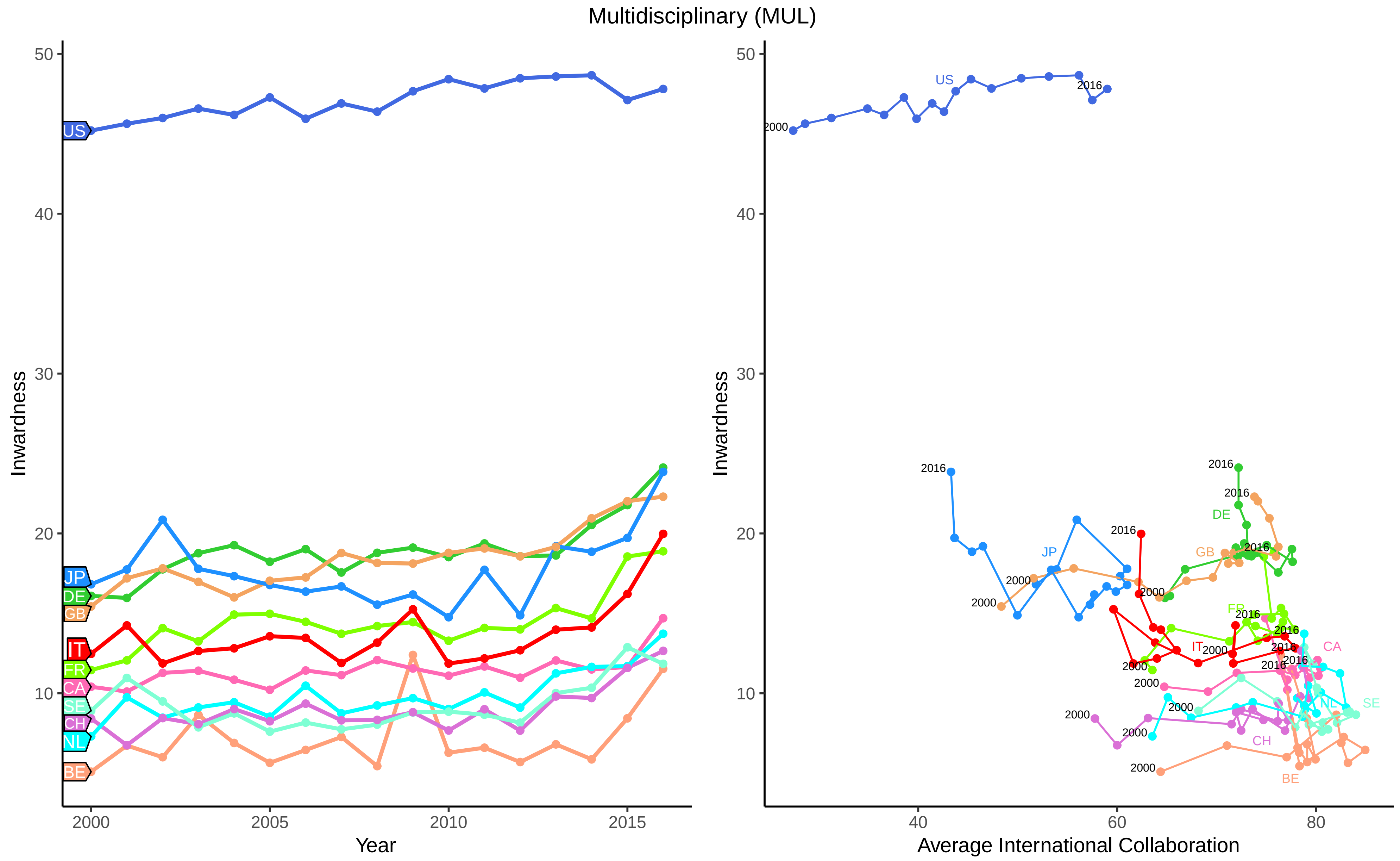

S1 Figure 20. Inwardness over time (left) and inwardness vs average international collaboration (right) for the G10 countries in Multidisciplinary (MUL)

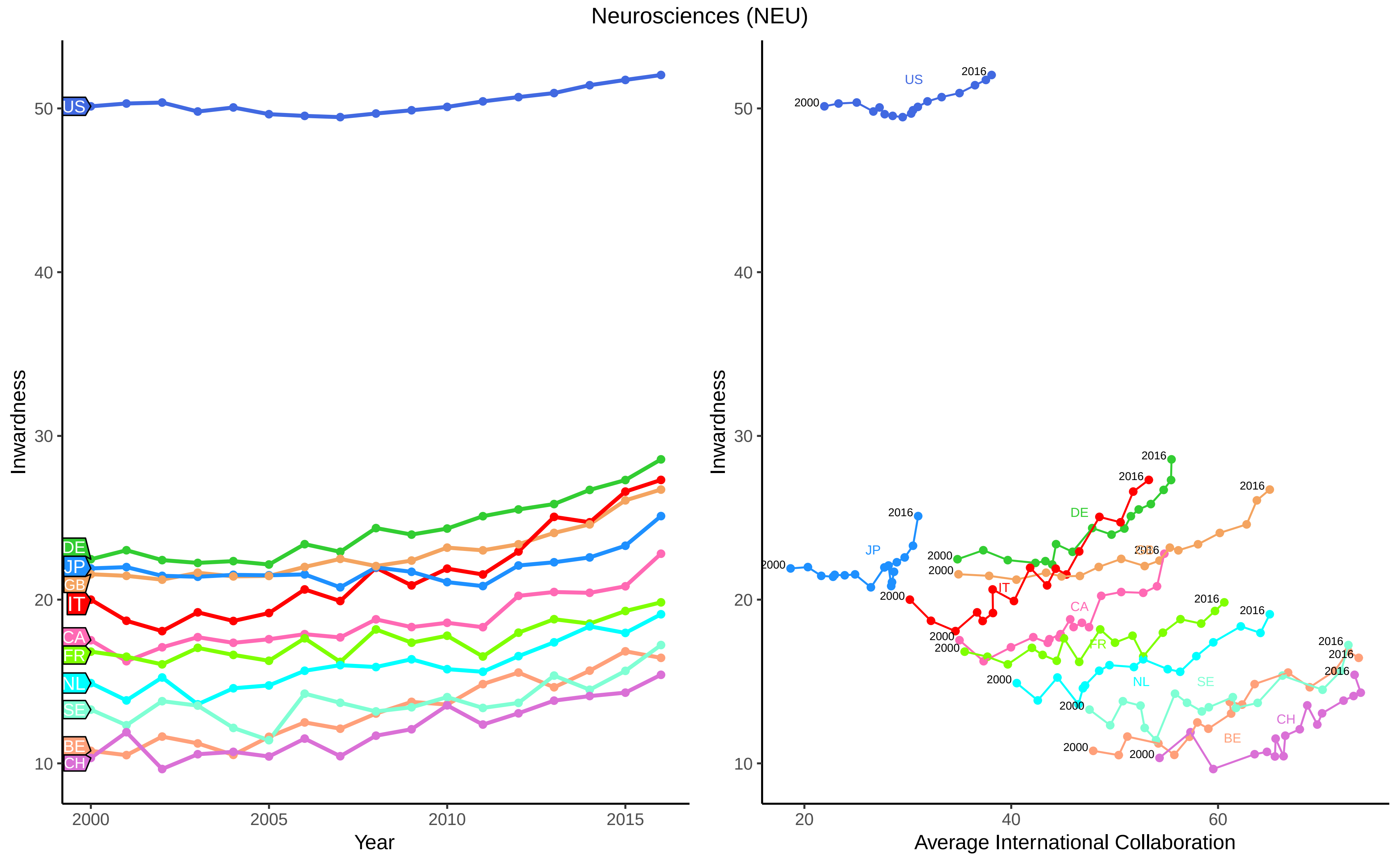

S1 Figure 21. Inwardness over time (left) and inwardness vs average international collaboration (right) for the G10 countries in Neurosciences (NEU)

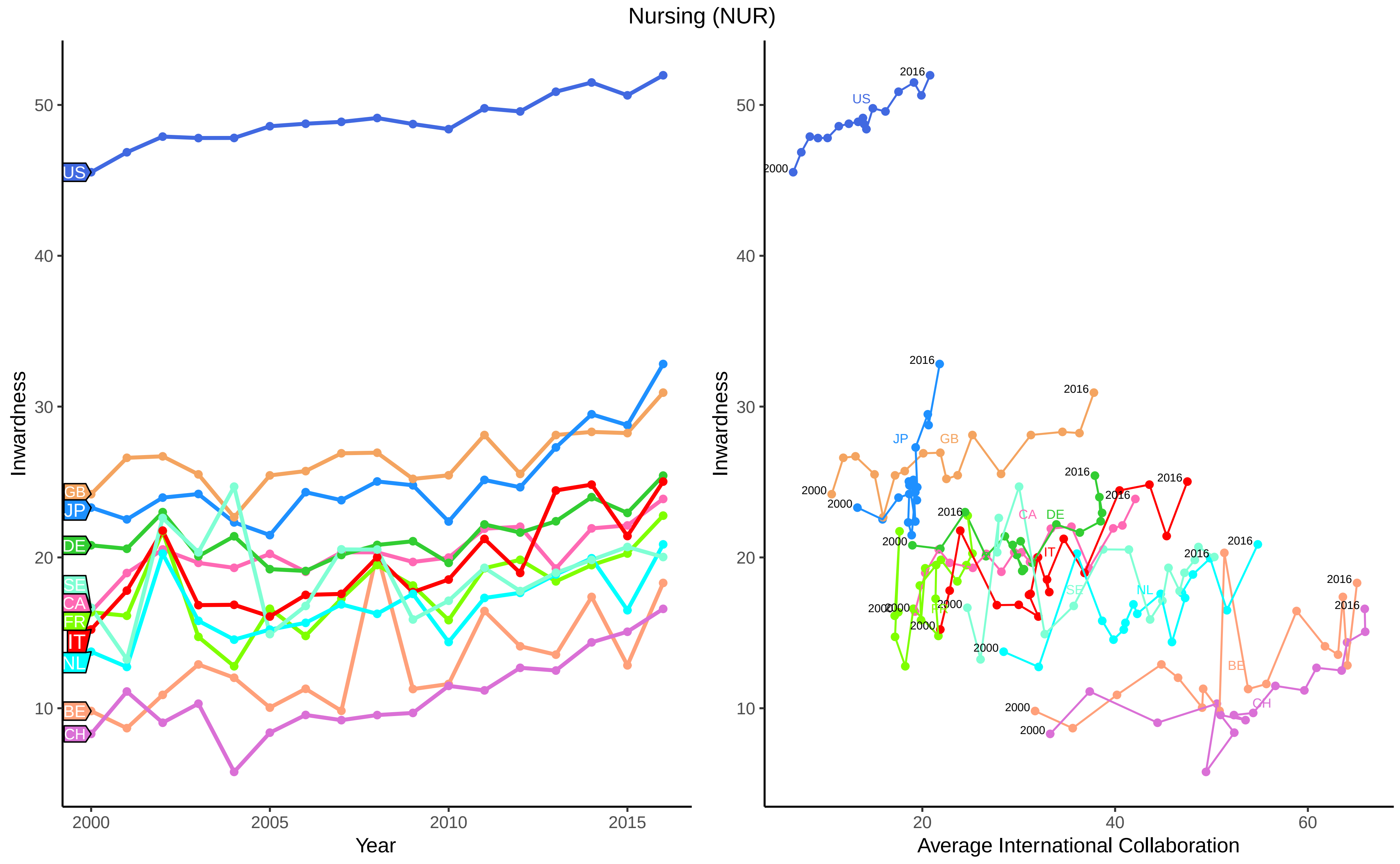

S1 Figure 22. Inwardness over time (left) and inwardness vs average international collaboration (right) for the G10 countries in Nursing (NUR)

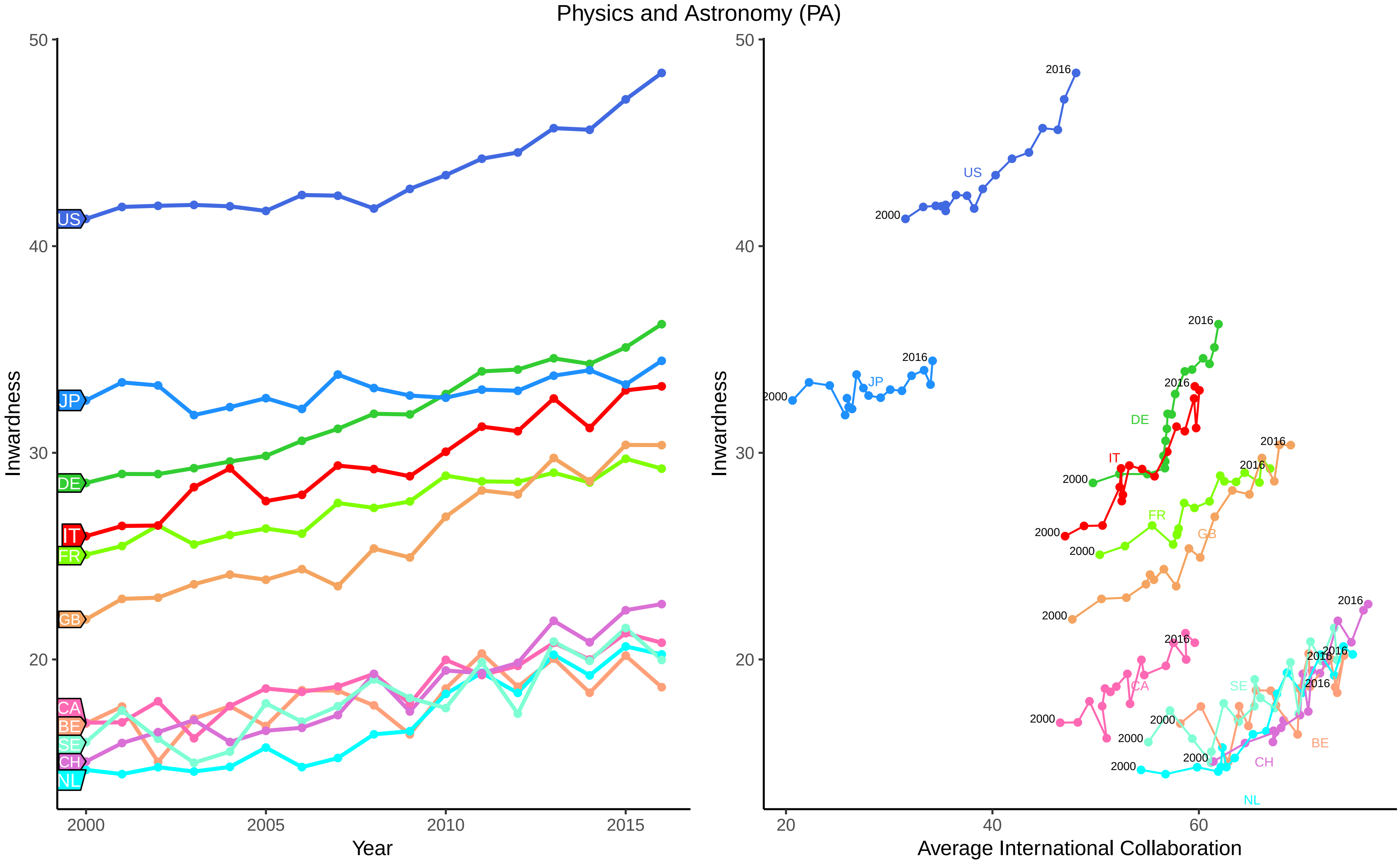

S1 Figure 23. Inwardness over time (left) and inwardness vs average international collaboration (right) for the G10 countries in Physics and Astronomy (PA)

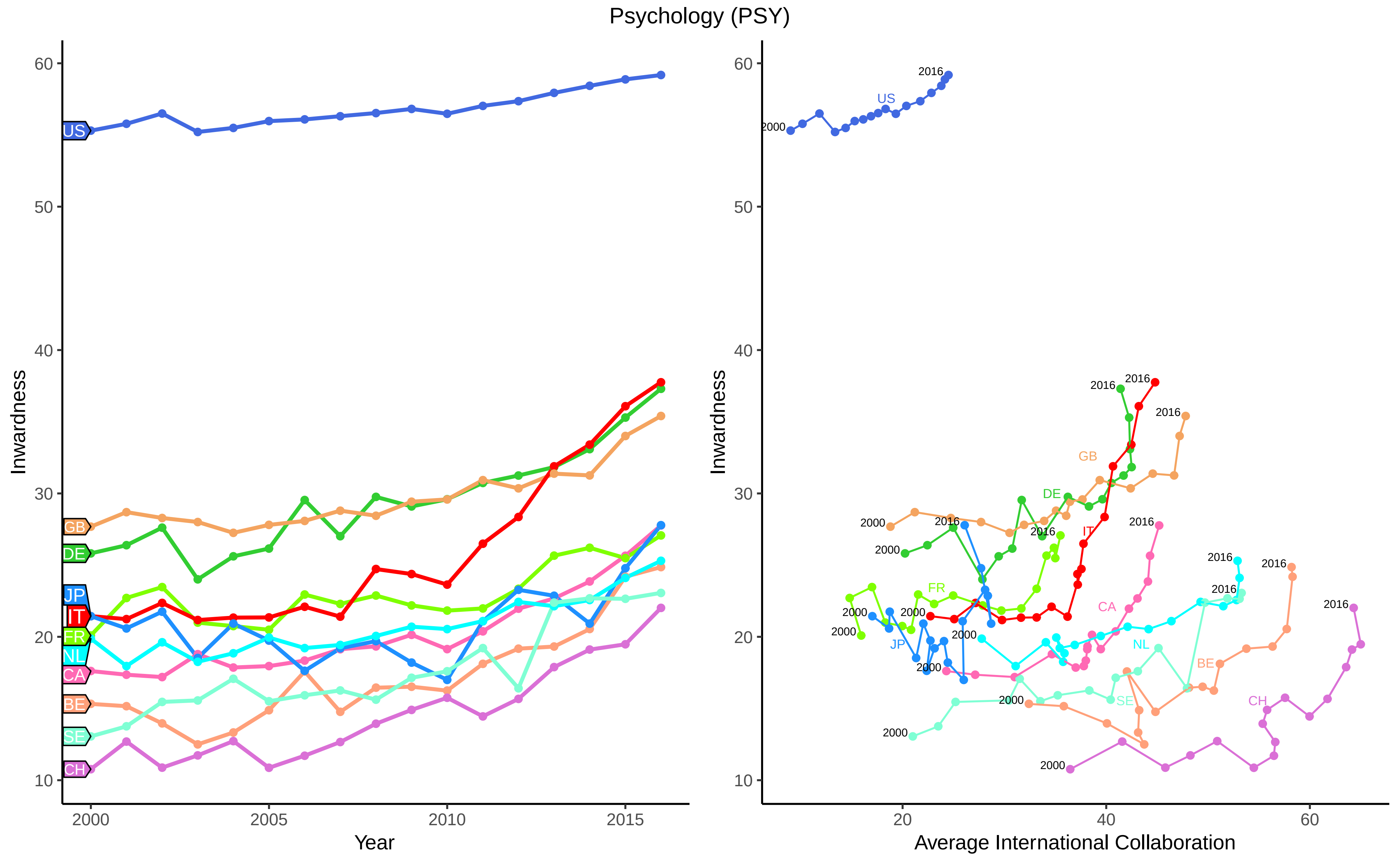

S1 Figure 24. Inwardness over time (left) and inwardness vs average international collaboration (right) for the G10 countries in Psychology (PSY)

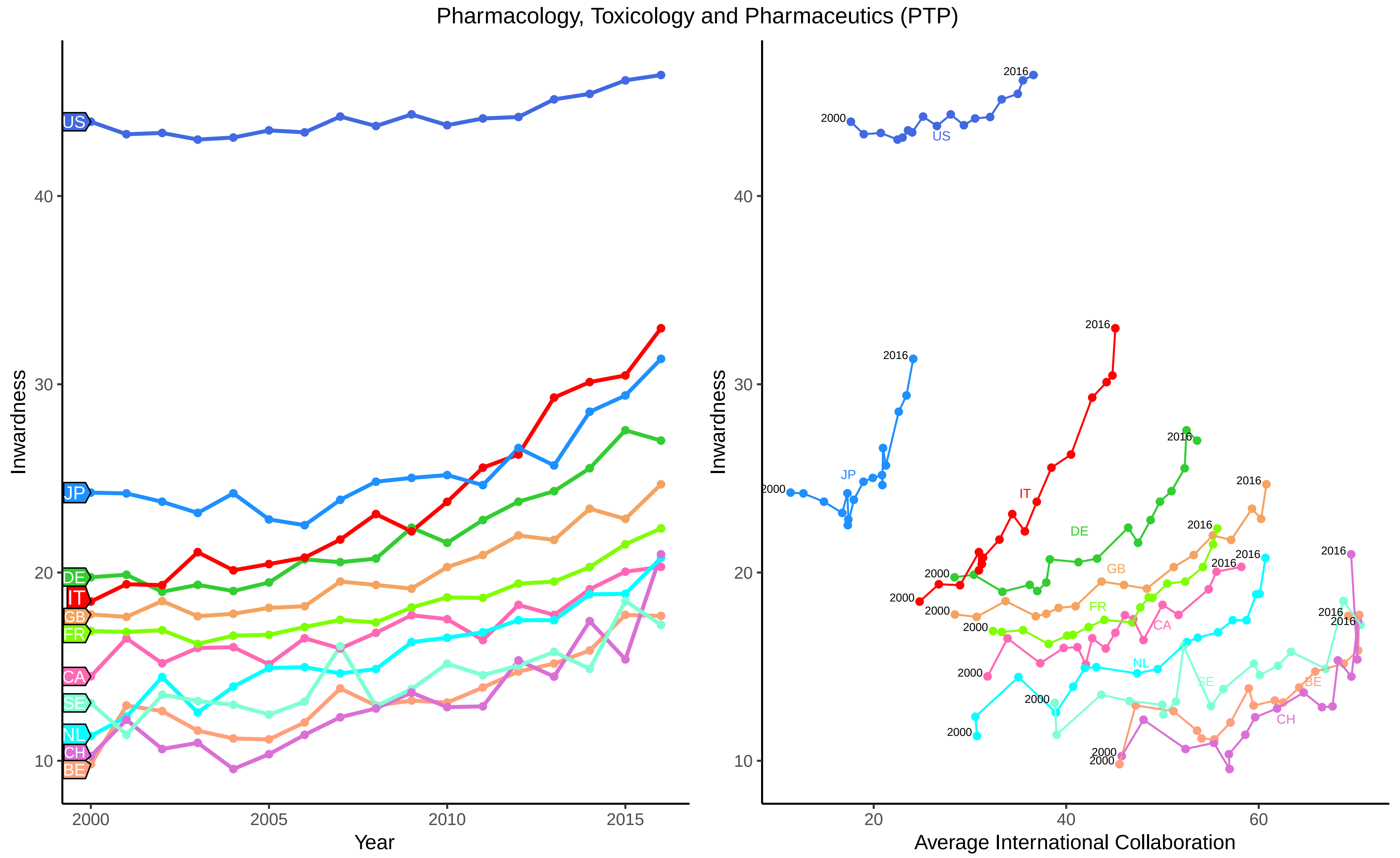

S1 Figure 25. Inwardness over time (left) and inwardness vs average international collaboration (right) for the G10 countries in Pharmacology, Toxicology and Pharmaceutics (PTP)

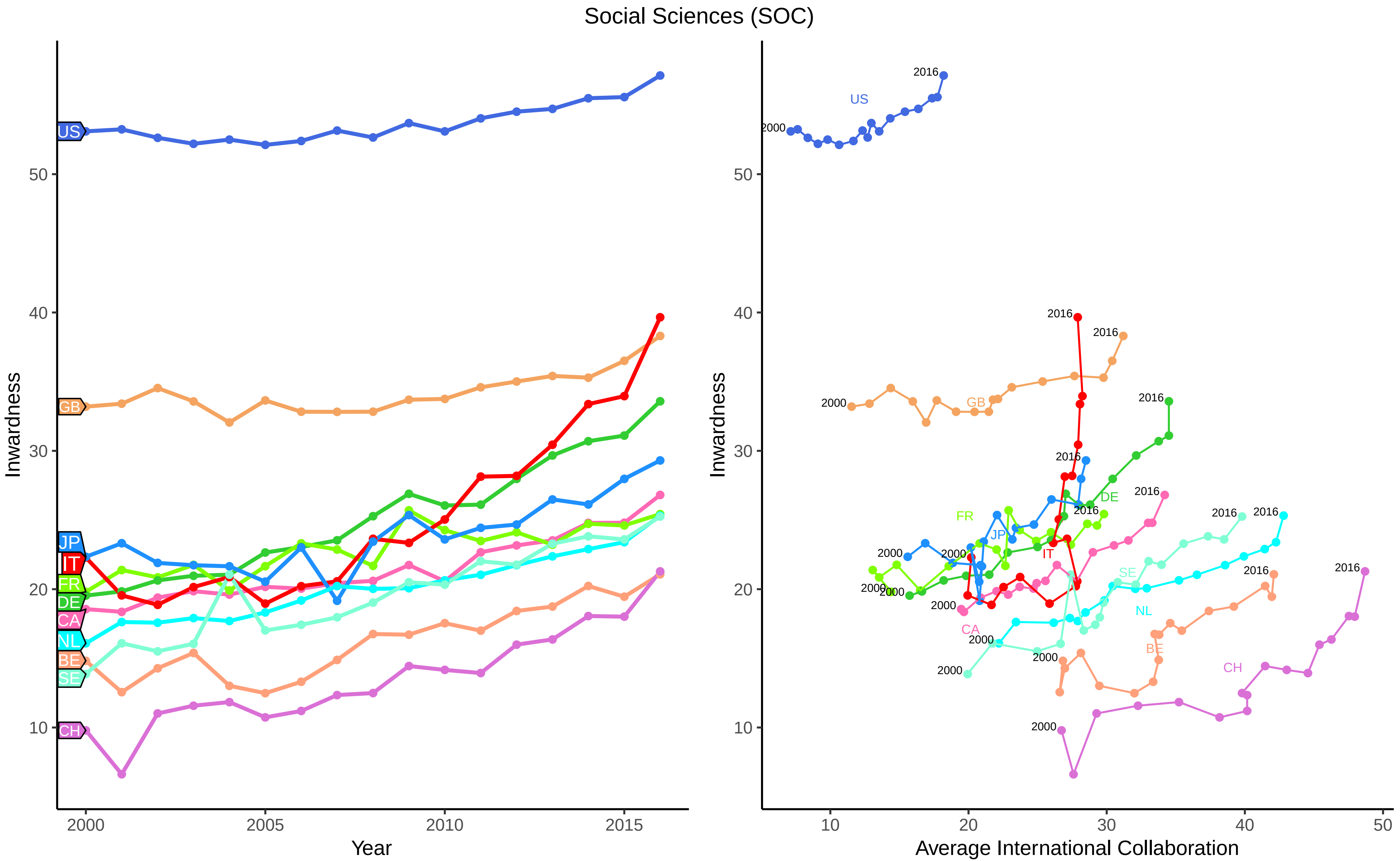

S1 Figure 26. Inwardness over time (left) and inwardness vs average international collaboration (right) for the G10 countries in Social Sciences (SOC)

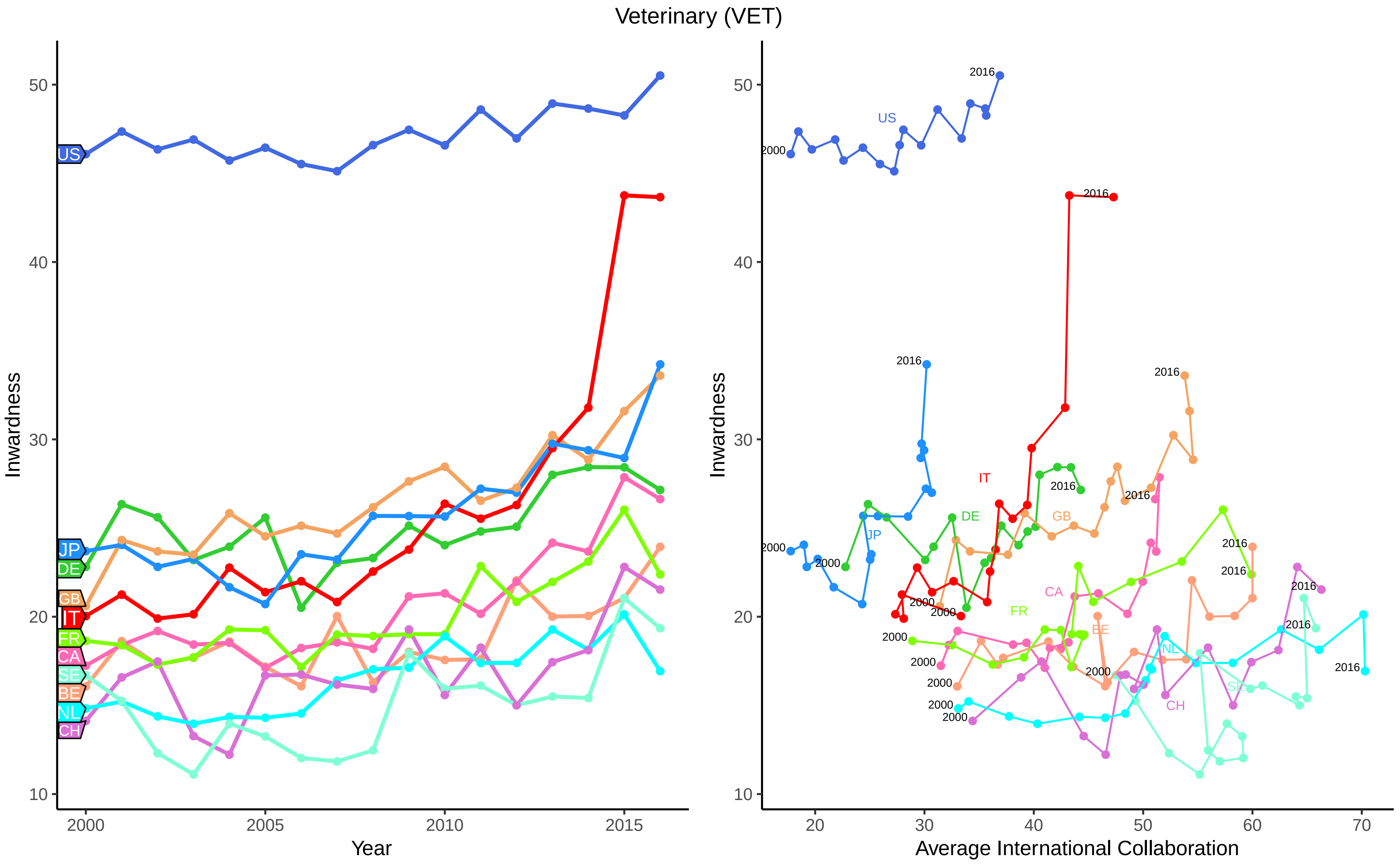

S1 Figure 27. Inwardness over time (left) and inwardness vs average international collaboration (right) for the G10 countries in Veterinary (VET)
